# Systematic Variation in Reviewer Practice According to Country and Gender in the Field of Ecology and Evolution

**DOI:** 10.1371/journal.pone.0003202

**Published:** 2008-09-12

**Authors:** Olyana N. Grod, Amber E. Budden, Tom Tregenza, Julia Koricheva, Roosa Leimu, Lonnie W. Aarssen, Christopher J. Lortie

**Affiliations:** 1 Department of Biology, York University, Toronto, Ontario, Canada; 2 National Center for Ecological Analysis and Synthesis (NCEAS), Santa Barbara, California, United States of America; 3 Centre for Ecology and Conservation, University of Exeter, Tremough, Penryn, United Kingdom; 4 School of Biological Sciences, Royal Holloway University of London, Egham, Surrey, United Kingdom; 5 Section of Ecology, University of Turku, Turku, Finland; 6 Department of Biology, Queens University, Kingston, Ontario, Canada; University of Aberdeen, United Kingdom

## Abstract

The characteristics of referees and the potential subsequent effects on the peer-review process are an important consideration for science since the integrity of the system depends on the appropriate evaluation of merit. In 2006, we conducted an online survey of 1334 ecologists and evolutionary biologists pertaining to the review process. Respondents were from Europe, North America and other regions of the world, with the majority from English first language countries. Women comprised a third of all respondents, consistent with their representation in the scientific academic community. Among respondents we found no correlation between the time typically taken over a review and the reported average rejection rate. On average, Europeans took longer over reviewing a manuscript than North Americans, and females took longer than males, but reviewed fewer manuscripts. Males recommended rejection of manuscripts more frequently than females, regardless of region. Hence, editors and potential authors should consider alternative sets of criteria, to what exists now, when selecting a panel of referees to potentially balance different tendencies by gender or region.

## Introduction

The peer-review process is an evaluation tool used to assess the merit of scientific work [1], [2]. Referees, experts in a particular field, are crucial to the success of the review system by providing impartial judgment on emerging research of their peers and colleagues [3]–[6]. They contribute many hours to the process, typically anonymously and with no remuneration [6]–[8]. Referees have a powerful influence on decisions made relating to publication [6], [9] and specific attributes associated with these individuals may relate to subjective manuscript evaluations.

A number of studies from various scientific disciplines have focused on the integrity of referees in assessing manuscripts and whether evaluations are based solely on the intrinsic quality of the manuscript or on factors unrelated to the research [1], [3], [10]–[12]. For instance, gender [10], [13], status [13] and an author's country of affiliation [11], [12] have been demonstrated to affect the referee recommendation to publish or reject a given manuscript [1], [3], [14]. This has been described as reviewer bias whereby the characteristics of an author are potentially used by referees and can influence manuscript acceptance [15]. However, few studies in ecology and evolution have looked explicitly at referee characteristics and how they relate to the review process. In disciplines such as medicine, it has been demonstrated that younger referees and those with more experience tend to score manuscripts lower [13]. Additionally, males have been shown to take longer to review, are more likely to ‘accept as is’, or are more likely to outright ‘reject’ relative to females in medicine [16]. Here, the importance of gender and scientific age of referee responses within ecology and evolution is similarly tested. Using an online survey, we assessed the importance of characteristics of ecological referees and their reported handling of manuscripts. We expected that ecology is similar to medicine in that gender, status, and region are important determinants of referee performance.

## Methods

### Design and Implementation of Survey

A web-based survey of ecologist and evolutionary biologists was designed by the National Centre for Ecological Analysis and Synthesis (NCEAS) Ecobias working group (www.ecobias.org), and was posted online from May 4^th^, 2006 to November 4^th^, 2006. A total of 17 questions relating to the publication process were included. For the purposes of this paper however, only those questions relevant to referee behaviour were tested and reported here (Text S1, Dataset S1, Dataset Notes S1). The questions were a combination of open-ended, multiple choice, and likert-scale questions. A group of high impact factor journals publishing ecology and evolutionary biology articles were listed. These were selected based on their 2004 impact factor. Nature, Science, PNAS and Current Biology were also included, as they are top biology journals even though not listed by ISI as ecology. We excluded those journals focusing on reviews (e.g. TREE, Annual Review of Ecology and Evolutionary Systematics) and specialty journals (e.g. Molecular Ecology, Global Change Biology). Despite only recent circulation, we included PLoS Biology which began in 2003 but was already receiving high citations. The final list comprised Nature, Science, Current Biology, PNAS, Ecological Monographs, American Naturalist, Ecology, Ecology Letters, Evolution and PLoS Biology. The survey was distributed to the Ecological Society of America (ECOLOG) and EvolDir mailing lists as well as promoted at international ecological and evolutionary conferences and posted on the working group website. These distribution lists were selected as a representative means to target ecologists and evolutionary biologists. The extent to which individual respondents subscribe to both list-serves was unknown hence the minimum (assuming there was complete overlap in subscribers to both list-serves) and maximum (where there was no subscription overlap) population sizes ranged from 6000 to 12 200. We received 1334 responses to the questionnaire, representing between 11% and 22% of the total population solicited.

As an estimate for experience, a potentially important covariate, the number of years involved in the publication process was estimated by subtracting the survey date from the reported year of first publication [17]. All countries were categorized to the following regions; North America, Europe, or ‘Other’. Official language designation was determined according to the country of host institution and characterized as English first language (EFL) or non-English first language (NEFL) using the United Nations Educational, Scientific and Cultural Organization classifications [18].

### Statistical Analyses

Chi-square analyses were used to describe the distribution of respondents according to their gender, region of affiliation, whether or not they published in or reviewed for the ‘top’ ecology journals, and referee language designation [19]. Generalized linear mixed models were used to test for an effect of gender and region on the number of manuscripts reviewed and reported review time. Due to the non-parametric distribution of some data, an ordinal logistic regression was used to test for effects of the above variables on individual reported rejection rates [19]. Tukey HSDs that control for multiple pair-wise tests were used for all post-hoc contrasts with the exception of the latter analysis where a post-hoc contingency table was used to test for differences between levels [19]. A logistic regression was used to test for the relationship between review time and rejection rate [20].

Years since first publication was treated as a covariate in all statistical analyses involving gender. All analyses were conducted with JMP®, Version 5.1 [21].

## Results

### Description of Respondent Population

**Figure 1:**
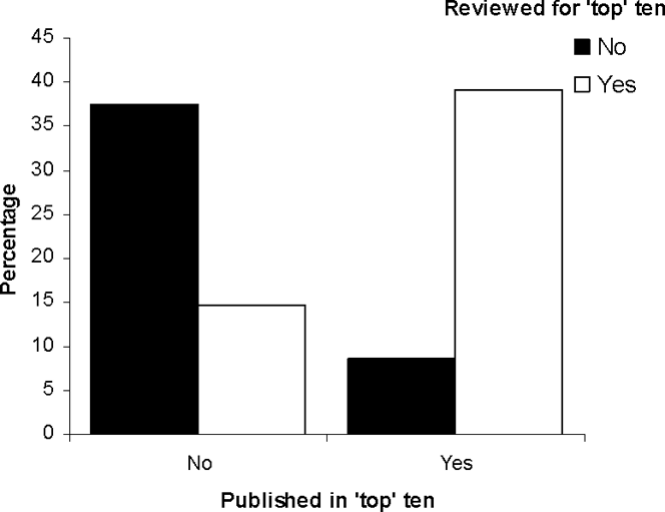
Respondent relationship between publishing and reviewing. Respondent publication and referee activity for the listed ‘top’ ten ecology journals (see text S1 for details).

There were significantly more male respondents (χ^2^_1_ = 156.18, p<0.001) with males representing 67.5% (n = 843) of respondents and females 32.5% (n = 406). North Americans comprised the dominant proportion of the respondent population with 60.5% (n = 752). Europeans comprised 32.3% (n = 401) and individuals from all ‘Other’ countries comprised 7.2% (n = 90); (χ^2^_2_ = 596.00, p<0.001; Figure 1). There was also significantly greater representation of EFL designated countries (χ^2^_1_ = 223.48, p<0.001). However, there was no difference between genders according to regions (χ^2^_2_ = 2.69, p = 0.261) or EFL designated countries (χ^2^_1_ = 1.34, p = 0.247).

Males published over significantly longer time periods than females (Mean_males_ = 10.96±0.42 SE, Mean_females_ = 7.87±0.53 SE years since first publication; F_1,5_ = 30.52, p<0.001). Overall, there was a significant difference in years since first publication among regions (F_2,5_ = 6.11 p = 0.002). North Americans had spent more years publishing than Europeans (10.62±0.31 and 8.66±0.43 years respectively; F_1,1190_ = 11.77, p<0.001). Respondents from Other regions did not differ significantly from North Americans (10.48±0.86 years; F_1,1190_ = 0.002, p = 0.964) nor from Europeans (F_1,1190_ = 3.43, p = 0.064). Since male ecologists had been actively publishing for longer, years since first publication was included as a covariate in subsequent analyses including gender.

The number of individuals that reviewed for the ten listed journals and those that did not was similar (χ^2^_1_ = 2.88, p = 0.090). However, fewer females (χ^2^_1_ = 25.65, p<0.001), and fewer respondents from NEFL designated countries (χ^2^_1_ = 23.46, p<0.001) reported reviewing for the listed journals. The referees for the listed journals spent significantly more years publishing than individuals who had not reviewed for these journals (12.97±0.29 vs 6.91±0.29 years ago; F_1,1278_ = 216.85 p<0.001) and spent significantly less time reviewing manuscripts on average (6.86±0.31 vs 7.98±0.33 hours; F_1,1165_ = 6.05 p = 0.014). The responses showed that if a respondent refereed for one of the listed journals they were more likely to have also published within such journals (χ^2^_1_ = 409.63, p<0.001; Figure 1).

### Handling of Manuscripts by Referees

**Figure 2:**
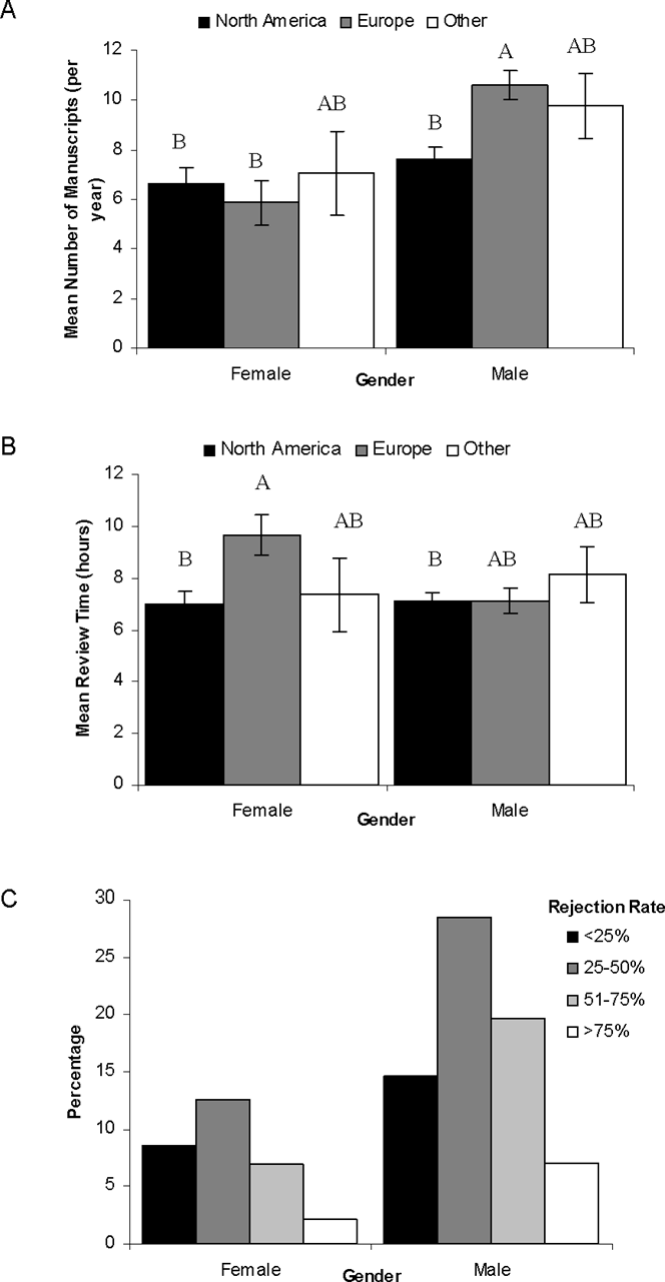
Relationships between referee gender and manuscript handling. Panel 2a shows the number of manuscripts reviewed per year, and 2b displays the time it takes to review a manuscript in hours. Data are presented as mean ±SE. Gender and regions not connected by the same letter are significantly different (Tukey HSD, p<0.05). Panel 2c highlights the rejection frequency among females and males.

Males reviewed significantly more manuscripts than women overall (9.13±0.50 vs 5.56±0.68 manuscripts per year; F_1,6_ = 11.06, p<0.001; Table 1). There was no difference between regions, however, there was a significant interaction between region and gender: European males reviewed significantly more manuscripts than European or North American females (F_2,6_ = 4.06, p = 0.018; Table 1; Figure 2a). There was no difference in review load of North American males and females (t_1,1074_ = 1.96 p = 0.192), or between referees from Other countries (p = 0.206). Although there appeared to be a difference between females from Other countries and females of North America and Europe, this was not significant after controlling for multiple comparisons. As expected, respondents who published over a longer period of time reviewed significantly more manuscripts (F_1,6_ = 137.36, p<0.001; Table 1).

**Table 1:** Relationship between manuscript handling and respondent demographics.

	F	p	Df_(effect, model)_
Number of manuscripts review per year			
Gender	11.06	**<0.001**	1, 6
Region	1.65	0.193	2, 6
Gender*Region	4.06	**0.018**	2, 6
Years since 1^st^ publication	137.36	**<0.001**	1, 6
Time spent reviewing (hours)			
Gender	0.58	0.446	1, 6
Region	3.07	**0.047**	2, 6
Gender*Region	3.29	**0.037**	2, 6
Years since 1^st^ publication	8.23	**0.004**	1, 6

	χ^2^	p	Df_(effect, model)_
Rejection rate			
Gender	3.74	**0.053**	1, 6
Region	2.02	0.365	2, 6
Gender*Region	1.85	0.398	2, 6
Years since 1^st^ publication	1.51	0.220	1, 6

General linear mixed models were used to test responses for respondent gender, region and the interaction of gender and region on the number of manuscripts reviewed and the time spent reviewing. An ordinal logistic regression was used to examine variation in reported rejection rates. In all cases, years since 1^st^ publication was included as a covariate (see text for details).

There was significant variation in the time spent reviewing according to region (F_2,6_ = 3.07, p = 0.047; Table 1) and Europeans took longer to review than North Americans (7.96±0.45 vs 7.00±0.32 hours). Again, there was a significant interaction between gender and region (F_2,6_ = 3.30, p = 0.037; Table 1) and European females spent more time reviewing than male or female North Americans (t_1,1078_ = 1.96 p = 0.001; Figure 2b). After controlling for multiple comparisons no significant difference appeared between European males and females. Similar to review load, males and females from Other countries (p = 0.665) and North American males and females (p = 0.840) did not differ in the time they invested in reviewing manuscripts. Respondents who had spent more years publishing spent significantly less time reviewing manuscripts (F_1,6_ = 8.23 p = 0.004).

Self-reported rates of rejection were higher for males than for females although only marginally significantly so (χ^2^ = 3.73, p = 0.053; Table 1). Post-hoc comparison of gender according to rejection rate showed that females ‘accepted’ (a self-reported rejection rate of <25%) significantly more manuscripts than males (χ^2^_3_ = 9.97, p = 0.019; Figure 2c). There was no effect of average time spent reviewing on the typical recommended decision regarding a manuscript (χ^2^_1_ = 2.11, p = 0.147).

## Discussion

Referees are integral to the peer-review process and are arguably a critical element that facilitates effective progress within a discipline. Therefore, a diverse and representative referee population with unique experiences and different scientific strengths promotes accurate and fair feedback on emerging scientific topics [3]. However, these potential strengths can also be a weakness if representation is uneven by gender or region, or if evaluation of a manuscript is based on factors that do not relate to the potential scientific merit of the work [2], [22]. Despite individual differences, more broad attributes of referees such as gender and region can act as determinants of a referee's handling of manuscripts, particularly in terms of the number of manuscripts being reviewed, review time and rejection rate. Editors need to consider the impact that referees individual traits can have on their evaluation of a manuscript and subsequent recommendation for publication.

The respondent population, a third of which was female, was representative of the general scientific community as documented by the National Science Foundation (NSF) and UNESCO, which independently reported that females comprised 30% of all academic science and engineering doctoral positions in the United States of America [23], and constituted 32% of scientific researchers in Europe [24]. Historically, men have been participating in science longer than women [25], and thus on average have more publishing experience. Our data showed that females had a lower average number of years since their first publication relative to males and we used this variable as a surrogate for activity within the publishing and refereeing process. In doing so, we presume that individuals have been actively participating in the peer-review process since the time of their first publication but we recognize that this may not always be the case, particularly for females who may take time off for childbearing. However, while the number of manuscripts reviewed and the time spent reviewing differed according to the time since first publication, there was no effect on the reported decision of a referee regarding manuscript rejection.

In addition to the appropriate representation of genders within our respondent population, there was diversity among regions, with respondents from countries outside of North America comprising over a third of the respondents, an unexpected response given that the survey was distributed through North American based list-serves. The sampling population was potentially bias as individuals who consult list-serves can have different characteristics than the bulk of the research community. We were unable to test for response bias as non-respondents could not be tracked due to the use of list-serves for survey distribution [17], [26].

Males reviewed significantly more papers than females. There are two possible explanations for this finding. First, it is possible that males are more likely to be asked to review by editors than females, either because there are more males in research or males are more preferred. Whether this is the case is uncertain and should be explored as females may represent a currently underutilized cohort within the ecological community. Second, males may be more likely to agree to review a manuscript if asked. However, it is probable that rates of both solicitation and acceptance affect the results obtained.

A gender region interaction for the number of manuscripts that are reviewed appears to be driven by a difference between European and males and females that is not present in other regions. Although it may appear that European males are more efficient referees, reviewing more papers than their female counterparts, the review times show that there was no difference in the amount of time European males and females spend reviewing manuscripts. The only significant difference in review time was for European females who take substantially longer than North American males and females. This was contrary to previous findings in medicine that male referees spend more time reviewing [16]. This difference might be explained by the size of each discipline. In medicine there may be a greater pool of available referees for manuscript review resulting in fewer requests per individual. Hence it is possible that medical referees are able to allocate more time per review than ecology and evolution referees who review more papers. Spending more or less time reviewing may reflect the degree of thoroughness but might also indicate referee efficiency. In this study, time spent reviewing did not correlate with typical rejection rate which also suggests that efficiency or degree of thoroughness for both positive and negative suggested outcomes is important.

There was no difference in the number of manuscripts reviewed or review time between North American males and females. The absence of a gender gap is promising; a sign that referees in North America are participating in the peer-review process to an equal extent. The survey also showed that gender, but not region of the referee, affected the recommendation to accept. This was consistent with previous work in which males assess manuscripts more strictly [13]. Thus having referees of all the same gender reviewing a manuscript can inappropriately increase or decrease the likelihood of a recommendation for publication.

The findings also have direct implications for referees who are in academia. Promotion in academia is often tied to the number of manuscripts a scholar has reviewed and the quality of the journals requesting reviews. Females, particularly European, are at a disadvantage in their probability for promotion, reviewing fewer manuscripts and fewer reviewing for ‘top’ ecology journals than males. Whether the composition of the editorial board affects the number of manuscripts reviewed by females and academics from NEFL countries needs to be considered.

Here, we demonstrate that referee gender and region can have implications for the way in which manuscripts are reviewed. The findings demonstrate that males and referees with greater publishing years review and reject more manuscripts than females and referees who started publishing more recently, and that Europeans spend the greatest amount of time reviewing. We propose that gender and geographical affiliation be considered by editors when recommending referees for the evaluation of manuscripts. Although the main criteria for choosing referees should be the extent of their expertise in a particular study area, where appropriate, these traits should be balanced. We recognize that ensuring such a balance becomes restrictive, however, editors should be cognizant of referee tendencies according to gender and region when evaluating their recommendations, and making a final decision for manuscript publication. In addition, the peer-review system would benefit from developing criteria for selecting referees and establishing more detailed standards for manuscript review [27]. Introduction of these measures will ensure that we control for the net effects of different referees on a given manuscript and generate more equitable reviews.

## Supporting Information

Text S1 Survey of the Peer Review and Publication Process in Ecology.
(0.02 MB PDF)

Dataset S1 Table of Responses to Survey of the Peer Review Process.
(0.05 MB PDF)

Dataset Notes S1 (0.01 MB PDF)
