# Supplementary material for: Systematic Variation in Reviewer Practice According to Country and Gender in the Field of Ecology and Evolution
Source: PLoS One. 2008 Sep 12;3(9):e3202. doi: 10.1371/journal.pone.0003202 (PMC2527679; doi:10.1371/journal.pone.0003202)
Supplement: Dataset Notes S1 — (0.01 MB PDF) [file pone.0003202.s003.pdf]

Notes to Dataset S1

Dataset only includes responses to survey questions of interest to this paper. Any unanswered questions were left blank.
