# Supplementary material for: Systematic Variation in Reviewer Practice According to Country and Gender in the Field of Ecology and Evolution
Source: PLoS One. 2008 Sep 12;3(9):e3202. doi: 10.1371/journal.pone.0003202 (PMC2527679; doi:10.1371/journal.pone.0003202)
Supplement: Text S1 — Survey of the Peer Review and Publication Process in Ecology. (0.02 MB PDF) [file pone.0003202.s001.pdf]

Text S1: Survey of the Peer Review and Publication Process in Ecology

(Questions of interest to this paper are marked in bold)

Many thanks for coming! This survey has 17 short questions. If you wish to leave at any time click 'exit this survey' (top right). Personal identifiers will not be used in any analyses and will remain confidential. Please answer all questions, if you don't want to answer a text question type NA or similar.

1. In which of the following journals have you published?

- Nature
- Science
- PNAS
- PLOS
- Current Biology
- Ecological Monographs
- American Naturalist
- Ecology
- Ecology Letters
- Evolution
- None of the above

2. Have you ever reviewed for any of the journals listed above?

Yes No

3. What proportion of the manuscripts that you review do you reject?

<25% 25-50% 51-75% >75% NA

4. What was the year of your first publication in a peer-reviewed journal? (Enter NA if not yet published)

5. Approximately how many manuscripts do you review for journals each year? (Enter NA if not applicable)

6. On average, how long do you spend reviewing a manuscript (in hours)? (Enter NA if not applicable)

7. When reviewing manuscripts, do you research unfamiliar authors using an internet search engine?

Never Sometimes Often Always Have not reviewed

8. Are you more likely to sign positive than negative reviews?

Yes No Decline to answer

9. Would you prefer to submit your manuscript to a journal where reviewers are not provided with the names of the authors?

Yes No No opinion

10. Would concealed author identity deter you from reviewing a manuscript?

Yes No No opinion

11. How would you describe your level of satisfaction with the review process as an author?

Highly satisfied Satisfied Neither satisfied nor dissatisfied Dissatisfied Highly dissatisfied

12. Rate the importance of the following factors when selecting a journal for submitting your manuscripts

	Very Important	Important	Somewhat Important	Not Important
High journal impact factor				
High likelihood of acceptance				
High likelihood of rapid decision				

13. Provide the reference of the paper that you consider to be your most significant contribution. Do not include invited or solicited papers. If you don't have the full reference, provide as much detail as possible. (Enter NA if you have not yet published)

14. How many times was this paper rejected from other journals before it was accepted for publication?
0 1 >1 NA

15. Was this paper rejected by either Nature or Science.
Yes No

16. What is your gender?
Female Male

17. In which country is your host institution?

Comments:
Email Address:

Many thanks for your time!
If you have any additional comments for the survey team regarding the publication process, please include them in the box below. You may also leave your email address if you wish to be updated on our work.
