# Supplementary material for: Systematic Variation in Reviewer Practice According to Country and Gender in the Field of Ecology and Evolution
Source: PLoS One. 2008 Sep 12;3(9):e3202. doi: 10.1371/journal.pone.0003202 (PMC2527679; doi:10.1371/journal.pone.0003202)
Supplement: Dataset S1 — Table of Responses to Survey of the Peer Review Process. (0.05 MB PDF) [file pone.0003202.s002.pdf]

Survey Complete?	Gender	Country	Region	Language	Reviewer?	Published in top 10?	Reviewed in top 10?	Rejection Rate	Years since 1st Publication	Number of Manuscripts Review (per year)	Review Time (hours)
Complete	Male	Canada	North America	English	Yes	Yes	Yes	>75%	10	15	1
Complete	Male	USA	North America	English	Yes	Yes	Yes	<25%	12	50	8
Complete	Male	USA	North America	English	Yes	No	No	>75%	1	2	5
Complete	Female	Canada	North America	English	Yes	No	Yes	25-50%	5	5	5
Complete	Female	USA	North America	English	No	No	No				
Complete	Male	USA	North America	English	Yes	No	No	<25%		2	12
Complete	Male	USA	North America	English	No	No	No		5		
Complete	Male	USA	North America	English	Yes	Yes	Yes	51-75%	9	6	2
Complete	Male	USA	North America	English	Yes	Yes	Yes	>75%	7	8	8
Complete	Male	USA	North America	English	Yes	Yes	Yes	25-50%	5	6	5
Complete	Male	Canada	North America	English	Yes	No	Yes	25-50%	13	15	3
Complete	Male	Brazil	Other	Non-English	Yes	No	No	25-50%	8	5	6
Complete	Female	USA	North America	English	Yes	Yes	Yes	<25%	31	12	1.5
Complete	Male	Canada	North America	English	Yes	Yes	Yes	>75%	8	5	4
Complete	Male	USA	North America	English	No	Yes	No		5		
Complete	Male	USA	North America	English	Yes	Yes	No	51-75%	9	12	3
Complete	Male	USA	North America	English	Yes	Yes	Yes	25-50%	23	4	14
Complete	Male	USA	North America	English	No	Yes	No		35		
Complete	Male	USA	North America	English	Yes	No	No	51-75%	12	1	6
Complete	Female	USA	North America	English	Yes	No	No	<25%	9	1	6
Complete	Female	USA	North America	English	Yes	Yes	Yes	51-75%	21	5	10
Incomplete					Yes	Yes	Yes	>75%	5	3	7
Complete	Male	USA	North America	English	Yes	Yes	Yes	51-75%	4	12	4
Complete	Male	USA	North America	English	Yes	No	No	25-50%	4	5	20
Complete	Female	USA	North America	English	No	No	No				
Complete	Male	USA	North America	English	Yes	Yes	Yes	51-75%	10	4	5
Complete	Male	USA	North America	English	Yes	No	Yes	25-50%	19	10	10
Complete	Female	USA	North America	English	Yes	Yes	No	<25%	5	3	6
Complete	Male	USA	North America	English	Yes	Yes	Yes	51-75%	20	10	6
Complete	Male	USA	North America	English	Yes	Yes	Yes	51-75%	5	1	25
Complete	Male	USA	North America	English	Yes	Yes	Yes	51-75%	3	6	5
Complete	Female	USA	North America	English	No	No	No		10		
Complete	Female	USA	North America	English	Yes	No	Yes	51-75%	1	4	4
Complete	Female	Mexico	North America	Non-English	Yes	No	No		9	3	6
Incomplete					No	No	No		5		
Complete	Male	USA	North America	English	Yes	No	Yes	25-50%	3	3	10
Incomplete					Yes	No	No	<25%	8	2	6
Complete	Male	USA	North America	English	Yes	No	No	<25%	3	2	8
Complete	Female	Australia	Other	English	Yes	No	No	51-75%	4	10	4
Incomplete					Yes	No	No	<25%	0	5	6

Complete	Male	USA	North America	English	Yes	No	No	<25%		5	5
Complete	Male	USA	North America	English	Yes	Yes	No	51-75%	8	3	5
Complete	Male	USA	North America	English	Yes	No	No	25-50%	3	0.5	4
Complete	Male	USA	North America	English	Yes	No	No	25-50%	2	4	3.5
Complete	Female	USA	North America	English	No	No	No		2		
Complete	Male	France	Europe	Non-English	Yes	No	No	25-50%	3	2	20
Complete	Female	USA	North America	English	Yes	No	No	51-75%	0	1	8
Incomplete					Yes	Yes	Yes	25-50%	4	2	4
Incomplete					Yes	No	Yes	25-50%	28	5	4
Complete	Male	USA	North America	English	Yes	Yes	Yes	>75%	9	5	7
Complete	Male	USA	North America	English	No	No	No				
Complete	Male	Sweden	Europe	Non-English	No	No	No		0		
Complete	Female	Finland	Europe	Non-English	Yes	Yes	Yes	25-50%	9	5	6
Complete	Male	Pakistan	Other	Non-English	No	No	No				
Complete	Male	UK	Europe	English	Yes	Yes	Yes	51-75%	26	20	5
Incomplete					Yes	Yes	Yes	>75%	9	30	9
Incomplete					Yes	No	Yes	>75%	4	3	10
Complete	Male	Norway	Europe	Non-English	Yes	No	No	25-50%	4	10	5
Complete	Male	Sweden	Europe	Non-English	Yes	Yes	Yes	25-50%	14	15	4
Complete	Female	Finland	Europe	Non-English	Yes	Yes	Yes	25-50%	11	12	18
Complete	Male	Denmark	Europe	Non-English	Yes	Yes	No	<25%	5	2	12
Complete	Female	Finland	Europe	Non-English	Yes	Yes	No	25-50%	10	1.5	6
Complete	Female	France	Europe	Non-English	Yes	No	No	<25%	4		10
Complete	Female	Finland	Europe	Non-English	Yes	No	No		5	2	5
Complete	Female	Netherlands	Europe	Non-English	No	No	No				
Complete	Male	Switzerland	Europe	Non-English	Yes	Yes	No	51-75%	4	6	10
Complete	Male	Finland	Europe	Non-English	Yes	No	No	51-75%	3	3	15
Complete	Male	Switzerland	Europe	Non-English	Yes	Yes	Yes	25-50%	7	10	7
Complete	Male	Poland	Europe	Non-English	Yes	Yes	Yes	51-75%	23	15	12
Complete	Female	France	Europe	Non-English	Yes	Yes	No	51-75%	9	5	4
Complete	Male	Austria	Europe	Non-English	Yes	No	No	<25%	12	4	6
Incomplete					Yes	Yes	No	<25%	7	4	6
Incomplete					Yes	Yes	Yes	25-50%	19	4	6
Complete	Male	Switzerland	Europe	Non-English	Yes	Yes	Yes	25-50%	9	7	4
Complete	Male	Germany	Europe	Non-English	Yes	Yes	No	25-50%	15	10	4
Complete	Female	Switzerland	Europe	Non-English	Yes	No	No		0		15
Complete	Male	Australia	Other	English	Yes	Yes	Yes	25-50%	10	4	4
Complete	Male	USA	North America	English	Yes	Yes	Yes	51-75%	11	8	3
Complete	Female	Germany	Europe	Non-English	Yes	No	No	<25%	2	3	6
Complete	Male	Italy	Europe	Non-English	Yes	No	No	25-50%	14	10	3
Complete	Female	Finland	Europe	Non-English	Yes	No	No	<25%	12	2	5
Incomplete					Yes	No	No	51-75%	5	2	16
Complete	Female	South Africa	Other	English	Yes	No	No	<25%	2	1	3
Complete	Female	Switzerland	Europe	Non-English	No	Yes	No		4		
Complete	Male	Switzerland	Europe	Non-English	Yes	Yes	Yes	<25%	12	15	8

Complete	Female	USA	North America	English	Yes	Yes	Yes	25-50%	12	6	4
Complete	Male	UK	Europe	English	Yes	Yes	Yes	25-50%	10	20	5
Complete	Male	Hungary	Europe	Non-English	Yes	No	No	<25%	7	4	3
Complete	Male	Germany	Europe	Non-English	Yes	No	No	<25%	2	2	5
Complete	Female	Slovenia	Europe	Non-English	Yes	No	No	25-50%	10	2	6
Complete	Female	USA	North America	English	Yes	Yes	Yes	25-50%	8	6	3
Complete	Male	Germany	Europe	Non-English	Yes	No	No	25-50%	3	2	5
Complete	Male	Germany	Europe	Non-English	Yes	Yes	Yes	25-50%	31	7	25
Complete	Male	Germany	Europe	Non-English	Yes	Yes	Yes	25-50%	14	40	3.5
Complete	Male	Switzerland	Europe	Non-English	Yes	Yes	No	<25%	5	2	5
Complete	Female	USA	North America	English	Yes	Yes	Yes	51-75%	7	12	3
Complete	Male	Germany	Europe	Non-English	Yes	Yes	Yes	>75%	11	25	4
Complete	Male	USA	North America	English	Yes	Yes	No	25-50%	4	2	1
Incomplete					Yes	Yes	No	25-50%	6	15	4
Complete	Female	France	Europe	Non-English	Yes	Yes	Yes	25-50%	12	1	
Complete	Male	Australia	Other	English	Yes	Yes	Yes	25-50%	12	50	1
Complete	Female	France	Europe	Non-English	Yes	No	No	25-50%	8	3	5
Complete	Male	UK	Europe	English	Yes	Yes	Yes	>75%	9	10	1
Incomplete					No	Yes	No		1		
Complete	Female	Australia	Other	English	Yes	No	Yes	25-50%	7	5	7
Complete	Female	Switzerland	Europe	Non-English	No	No	No		4		
Complete	Male	Sweden	Europe	Non-English	Yes	Yes	Yes	51-75%	18	100	3
Complete	Male	Sweden	Europe	Non-English	Yes	Yes	Yes	51-75%	14	17.5	8
Complete	Male	Germany	Europe	Non-English	Yes	Yes	Yes	25-50%	14	12.5	6
Complete	Female	Sweden	Europe	Non-English	No	No	No		1		
Incomplete					Yes	Yes	Yes	25-50%	6	2	5
Incomplete					Yes	Yes	Yes	25-50%	8	20	10
Complete	Male	Netherlands	Europe	Non-English	Yes	Yes	Yes	>75%	12	50	5
Complete	Female	Finland	Europe	Non-English	Yes	Yes	Yes	>75%	7	2	8
Complete	Male	Netherlands	Europe	Non-English	Yes	Yes	No	51-75%	2	8	5
Complete	Male	Switzerland	Europe	Non-English	Yes	Yes	Yes	51-75%	9	10	3
Complete	Male	UK	Europe	English	Yes	Yes	No	25-50%	3	5	4
Complete	Female	France	Europe	Non-English	No	No	No		2		
Complete	Male	Switzerland	Europe	Non-English	Yes	No	No	51-75%	7	8	3
Complete	Male	Sweden	Europe	Non-English	Yes	Yes	Yes	51-75%	6	1	24
Complete	Male	Sweden	Europe	Non-English	Yes	Yes	Yes	25-50%	12	12	16
Complete	Male	Switzerland	Europe	Non-English	Yes	Yes	Yes	51-75%	7	17	5
Complete	Male	France	Europe	Non-English	Yes	No	No		24	3	2
Complete	Female	France	Europe	Non-English	No	No	No		3		
Complete	Male	UK	Europe	English	Yes	Yes	Yes	25-50%	13	10	6
Complete	Male	Netherlands	Europe	Non-English	No	No	No		2		
Complete	Male	Sweden	Europe	Non-English	Yes	No	No	>75%	0	1	20
Complete	Male	Germany	Europe	Non-English	Yes	Yes	Yes	51-75%	21	50	4
Complete	Male	Germany	Europe	Non-English	Yes	No	No	>75%	7	5	4
Complete	Female	France	Europe	Non-English	Yes	No	No	51-75%	16	10	2.5

Complete	Male	Sweden	Europe	Non-English	Yes	Yes	No		7	1	8
Complete	Female	New Zealand	Other	English	Yes	Yes	Yes	25-50%	10	5	5
Complete	Male	Germany	Europe	Non-English	No	No	No		1		
Complete	Female	Austria	Europe	Non-English	Yes	No	Yes	25-50%	3	4	16
Complete	Male	Germany	Europe	Non-English	Yes	No	Yes	<25%	8	10	5
Complete	Male	UK	Europe	English	Yes	Yes	Yes	25-50%	20	50	2
Complete	Male	Germany	Europe	Non-English	Yes	Yes	Yes	<25%	5	3	16
Complete	Male	Norway	Europe	Non-English	Yes	Yes	Yes	25-50%	14	20	8
Complete	Male	UK	Europe	English	Yes	Yes	Yes	25-50%	11	15	2
Complete	Male	UK	Europe	English	Yes	Yes	Yes	51-75%	10	15	5
Complete	Female	Austria	Europe	Non-English	Yes	No	No	<25%	9	5	10
Complete	Male	Finland	Europe	Non-English	Yes	Yes	Yes	51-75%	10	10	4
Complete	Male	Belgium	Europe	Non-English	Yes	No	No	25-50%	2	10	6
Complete	Male	Spain	Europe	Non-English	Yes	Yes	Yes	51-75%	15	3	6
Complete	Male	New Zealand	Other	English	Yes	Yes	Yes	25-50%	20	10	5
Complete	Female	France	Europe	Non-English	Yes	No	No	<25%	8	2	24
Incomplete					Yes	Yes	Yes	>75%	5	12	6
Complete	Male	Germany	Europe	Non-English	Yes	Yes	No	51-75%	9	3	5
Complete	Female	Switzerland	Europe	Non-English	Yes	Yes	Yes	51-75%	10	12	8
Complete	Male	Australia	Other	English	Yes	No	Yes	25-50%	21	25	1.5
Complete	Male	Germany	Europe	Non-English	Yes	No	No	<25%	5	1	12
Complete	Female	Australia	Other	English	Yes	Yes	No	>75%	3	12	6
Complete	Male	UK	Europe	English	Yes	Yes	No	25-50%	3	10	5
Incomplete					No	No	No		1		
Complete	Female	Spain	Europe	Non-English	Yes	Yes	No	25-50%	14	1.5	9.5
Complete	Male	Sweden	Europe	Non-English	Yes	Yes	Yes	51-75%	13	25	1
Complete	Male	Belgium	Europe	Non-English	Yes	Yes	No	25-50%	13	5	5
Complete	Male	UK	Europe	English	Yes	Yes	Yes	<25%	22	50	4
Complete	Male	Germany	Europe	Non-English	Yes	No	No	51-75%	7	15	7
Complete	Male	India	Other	Non-English	Yes	Yes	Yes	25-50%	18	6	5
Complete	Male	Germany	Europe	Non-English	Yes	Yes	Yes	51-75%	13	15	8
Complete	Male	UK	Europe	English	Yes	Yes	No	25-50%	6	2	6
Complete	Female	South Africa	Other	English	Yes	No	No	51-75%	1	3	8
Complete	Female	South Africa	Other	English	Yes	Yes	No	51-75%	8	12	8
Incomplete					Yes	Yes	Yes	25-50%	8	14	2
Complete	Male	Germany	Europe	Non-English	Yes	Yes	No	25-50%	13	5	10
Incomplete					No	No	No		3		
Complete	Male	Australia	Other	English	Yes	Yes	Yes	25-50%	14	20	2
Complete	Female	Italy	Europe	Non-English	Yes	Yes	Yes	25-50%	9	3	72
Complete	Female	Netherlands	Europe	Non-English	Yes	Yes	No		5	1	6
Complete	Male	France	Europe	Non-English	Yes	Yes	No	51-75%	10	5	10
Complete	Male	Australia	Other	English	Yes	Yes	Yes	51-75%	4	10	6
Complete	Male	Portugal	Europe	Non-English	No	No	No		4		
Complete	Female	France	Europe	Non-English	Yes	Yes	No	25-50%	10	15	2.5
Complete	Male	UK	Europe	English	Yes	Yes	Yes	<25%	9	6	3

Complete	Female	UK	Europe	English	Yes	Yes	No	25-50%	3	2	6
Complete	Female	UK	Europe	English	Yes	No	No	25-50%	4	1	4
Complete	Male	Switzerland	Europe	Non-English	Yes	Yes	No	25-50%	9	8	6
Complete	Female	UK	Europe	English	Yes	Yes	Yes	25-50%	5	8	6
Complete	Male	UK	Europe	English	Yes	Yes	Yes	>75%	12	20	4
Complete	Male	USA	North America	English	No	No	No				
Complete	Female	UK	Europe	English	Yes	No	No	<25%	6	1	6
Complete	Female	France	Europe	Non-English	Yes	No	No	25-50%	3	2	20
Complete	Female	France	Europe	Non-English	Yes	No	No	25-50%	6	0.3	10
Complete	Male	France	Europe	Non-English	Yes	No	No	25-50%	4	5	6
Incomplete					Yes	No	Yes		10	2	4
Complete	Male	UK	Europe	English	Yes	No	No	<25%	6	1	12
Complete	Male	France	Europe	Non-English	No	No	No		2		
Complete	Male	Austria	Europe	Non-English	Yes	Yes	No	25-50%	8	10	6
Complete	Male	Switzerland	Europe	Non-English	Yes	No	No	51-75%	7	20	4
Complete	Female	Denmark	Europe	Non-English	Yes	Yes	No	<25%	3	4.5	7
Complete	Male	France	Europe	Non-English	Yes	Yes	Yes	<25%	6	2	15
Complete	Male	Germany	Europe	Non-English	Yes	No	No	25-50%	4	3	4
Complete	Male	Australia	Other	English	Yes	Yes	Yes	51-75%	23	35	2.5
Complete	Female	Australia	Other	English	Yes	No	No	<25%	8	0.5	4
Complete	Male	Germany	Europe	Non-English	Yes	Yes	Yes	>75%	8	10	4
Complete	Female	Germany	Europe	Non-English	Yes	No	Yes	25-50%	8	15	5
Complete	Male	UK	Europe	English	Yes	Yes	Yes	25-50%	10	4	3
Complete	Male	Australia	Other	English	Yes	No	No	<25%	5	6	10
Complete	Female	Italy	Europe	Non-English	Yes	Yes	No	<25%	4	2	8
Complete	Female	Australia	Other	English	No	No	No		2		
Complete	Male	Germany	Europe	Non-English	Yes	No	No	51-75%	2	2	10
Complete	Male	USA	North America	English	Yes	No	No	>75%	13	6	20
Complete	Male	South Africa	Other	English	Yes	Yes	No	<25%	22	3	4
Complete	Male	USA	North America	English	Yes	No	No	25-50%	12	0.5	25
Complete	Female	Germany	Europe	Non-English	Yes	No	No	<25%	2	1	20
Complete	Male	Canada	North America	English	Yes	Yes	Yes	25-50%	3	6	2
Incomplete					Yes	Yes	No	25-50%	4	5	1
Complete	Male	Sweden	Europe	Non-English	Yes	Yes	Yes	51-75%	5	3	16
Complete	Male	Portugal	Europe	Non-English	Yes	Yes	Yes	<25%	6	30	2
Complete	Female	UK	Europe	English	Yes	Yes	Yes	25-50%	3	2	3
Incomplete					No	No	No		1		
Complete	Male	Sweden	Europe	Non-English	Yes	Yes	Yes	25-50%	6	10	6
Complete	Male	USA	North America	English	Yes	No	No	<25%	36	5	10
Complete	Male	UK	Europe	English	Yes	Yes	No	<25%	5	2	3
Complete	Female	Germany	Europe	Non-English	No	No	No		1		
Complete	Female	UK	Europe	English	Yes	No	Yes	25-50%	6	4	7
Incomplete					No	No	No				
Complete	Male	Germany	Europe	Non-English	Yes	Yes	No	51-75%	3	2	5
Complete	Male	Netherlands	Europe	Non-English	Yes	Yes	Yes	25-50%	23	40	3

Complete	Male	UK	Europe	English	Yes	Yes	No	<25%	2	1	4
Complete	Male	Germany	Europe	Non-English	Yes	Yes	Yes	25-50%	30	30	6
Complete	Female	Italy	Europe	Non-English	Yes	No	No	25-50%	14	5	24
Complete	Male	Israel	Other	Non-English	Yes	No	No	51-75%	0	4	6
Complete	Male	Switzerland	Europe	Non-English	Yes	No	No	25-50%	5	4.5	7.5
Complete	Female	USA	North America	English	Yes	Yes	Yes	51-75%	10	4	48
Complete	Female	UK	Europe	English	Yes	Yes	Yes	<25%	5	6	5
Complete	Male	Finland	Europe	Non-English	Yes	Yes	Yes	51-75%	28	30	6
Complete	Male	UK	Europe	English	Yes	Yes	Yes	25-50%	31	20	1.5
Complete	Female	USA	North America	English	Yes	Yes	Yes	25-50%	7	10	4
Complete	Male	Ireland	Europe	English	Yes	No	No	51-75%	4	5	12
Incomplete					Yes	Yes	No	<25%	20	8	6
Complete	Male	Sweden	Europe	Non-English	Yes	Yes	Yes	<25%	18	10	6
Complete	Female	Netherlands	Europe	Non-English	Yes	No	No	<25%	4	3	5
Complete	Male	Netherlands	Europe	Non-English	Yes	No	No		2	1	5
Complete	Male	Italy	Europe	Non-English	Yes	Yes	No		6	1	5
Incomplete					Yes	Yes	Yes	<25%	8	6	4
Complete	Female	Sweden	Europe	Non-English	Yes	Yes	Yes	51-75%	23	6	7
Complete	Male	USA	North America	English	Yes	No	No	<25%	8	6	15
Complete	Male	USA	North America	English	Yes	No	No	25-50%	24	5	1
Complete	Male	France	Europe	Non-English	Yes	No	No	51-75%	16	4	6
Complete	Male	USA	North America	English	Yes	Yes	Yes	25-50%	9	4	4
Complete	Male	USA	North America	English	Yes	Yes	Yes	25-50%	17	50	3
Complete	Female	USA	North America	English	Yes	No	No	51-75%	10	8	8
Complete	Male	UK	Europe	English	Yes	Yes	Yes	51-75%	8	3	3
Complete	Male	USA	North America	English	Yes	No	No	25-50%	4	2	6
Incomplete					No	Yes	No		4		
Complete	Female	USA	North America	English	Yes	No	No	51-75%	8	3	12.5
Complete	Male	Denmark	Europe	Non-English	Yes	Yes	Yes	25-50%	3	5	5
Complete	Female	South Africa	Other	English	No	No	No				
Complete	Female	USA	North America	English	Yes	Yes	Yes	51-75%	17	40	3.5
Complete	Female	UK	Europe	English	Yes	No	No	<25%	6	5	8
Complete	Male	Ireland	Europe	English	Yes	Yes	Yes	25-50%	11	10	2
Complete	Male	Canada	North America	English	Yes	No	No	25-50%	5	3	4
Complete	Male	Canada	North America	English	Yes	Yes	Yes	51-75%	3	12	5
Complete	Female	South Africa	Other	English	Yes	No	No	<25%	5	2	8
Complete	Male	USA	North America	English	Yes	Yes	Yes	51-75%	16	15	3
Complete	Male	USA	North America	English	Yes	Yes	Yes	25-50%	19	14	4
Complete	Male	Australia	Other	English	Yes	Yes	No		3	1	5
Complete	Male	Switzerland	Europe	Non-English	Yes	No	No	<25%	13	2	2
Complete	Male	UK	Europe	English	No	No	No		1		
Complete	Female	UK	Europe	English	Yes	No	Yes	<25%	5	10	5
Complete	Male	Canada	North America	English	Yes	Yes	Yes	25-50%	9	2	8
Complete	Male	Germany	Europe	Non-English	Yes	Yes	No	25-50%	4	5	4
Incomplete					No	Yes	No		5		

Complete	Male	USA	North America	English	Yes	No	No	<25%	7	3	8
Complete	Female	South Africa	Other	English	Yes	Yes	Yes	25-50%	34	8	6
Complete	Male	UK	Europe	English	Yes	Yes	Yes	25-50%	19	16	2
Complete	Male	Canada	North America	English	Yes	Yes	Yes	>75%	16	60	2
Complete	Female	France	Europe	Non-English	Yes	No	No	<25%		3	20
Complete	Male	Sweden	Europe	Non-English	No	No	No		8		
Complete	Male	Canada	North America	English	Yes	No	No	<25%	3	6	2
Incomplete					Yes	Yes	Yes	51-75%	12		
Complete	Male	USA	North America	English	No	Yes	No		1		
Complete	Male	South Africa	Other	English	Yes	No	Yes	25-50%			
Complete	Male	USA	North America	English	Yes	No	No	<25%	8	1	3
Complete	Male	USA	North America	English	Yes	Yes	Yes	51-75%	10	6	8
Complete	Male	South Africa	Other	English	Yes	No	No	25-50%	9	2	8
Complete	Male	USA	North America	English	Yes	Yes	No	51-75%	1	2	3
Complete	Male	USA	North America	English	Yes	Yes	Yes	<25%	15	6	2.5
Complete	Male	Belgium	Europe	Non-English	No	No	No		2		
Complete	Female	USA	North America	English	Yes	No	No		5	1	6
Complete	Male	UK	Europe	English	Yes	Yes	No	51-75%	5	4	14
Complete	Male	USA	North America	English	Yes	Yes	Yes	51-75%	29	20	5
Incomplete					Yes	No	No	<25%	12	2	15
Complete	Male	South Africa	Other	English	No	No	No				
Complete	Female	Portugal	Europe	Non-English	Yes	No	No	<25%	8	1	8
Complete	Male	Ireland	Europe	English	No	No	No		2		
Complete	Male	UK	Europe	English	Yes	No	Yes	>75%	2	3	12
Complete	Male	UK	Europe	English	Yes	Yes	Yes	51-75%	14	25	4
Complete	Male	USA	North America	English	Yes	Yes	Yes	25-50%	34	15	6
Complete	Female	USA	North America	English	Yes	Yes	No	25-50%	3	1	5
Complete	Female	USA	North America	English	Yes	Yes	Yes	25-50%	14	5	8
Complete	Male	France	Europe	Non-English	Yes	Yes	Yes	51-75%	5	25	6
Complete	Male	USA	North America	English	Yes	Yes	Yes	51-75%	10	6	6
Complete	Female	USA	North America	English	Yes	No	No	<25%	4	3	10
Complete	Male	UK	Europe	English	Yes	No	No		9	0.5	8
Complete	Male	USA	North America	English	Yes	Yes	Yes	51-75%	11	25	2
Complete	Male	USA	North America	English	Yes	Yes	Yes	<25%	5	10	2
Incomplete					No	No	No		1		
Complete	Female	Israel	Other	Non-English	Yes	No	No	>75%	2	3.5	6
Incomplete					Yes	Yes	No	<25%	5	8	10
Complete	Female	UK	Europe	English	Yes	No	No	<25%	4	4	6
Complete	Male	USA	North America	English	Yes	Yes	Yes	25-50%	10	12	5
Complete	Female	USA	North America	English	Yes	No	No				
Complete	Male	USA	North America	English	No	No	No		1		
Complete	Male	USA	North America	English	Yes	Yes	Yes	25-50%	7	10	4
Complete	Female	USA	North America	English	Yes	Yes	No		3	4	10
Complete	Male	Canada	North America	English	Yes	Yes	Yes	<25%	21	4	8
Complete	Male	USA	North America	English	Yes	No	Yes	25-50%	10	2	10

Complete	Male	USA	North America	English	Yes	No	No	25-50%	7	9	24
Complete	Male	USA	North America	English	Yes	Yes	Yes	<25%	7	12	8
Complete	Male	Canada	North America	English	Yes	Yes	No	<25%	39	10	2
Complete	Male	USA	North America	English	Yes	Yes	Yes	<25%	3	4	8
Complete	Male	Germany	Europe	Non-English	No	Yes	No		0		
Complete	Female	Argentina	Other	Non-English	Yes	Yes	No	25-50%	10	1	40
Complete	Male	Germany	Europe	Non-English	Yes	No	Yes	>75%	6	10	2
Complete	Male	Netherlands	Europe	Non-English	Yes	Yes	No	25-50%	5	3	6
Complete	Male	Belgium	Europe	Non-English	Yes	Yes	Yes	<25%	7	15	6
Complete	Female	USA	North America	English	Yes	Yes	Yes	51-75%	7	5	6
Complete	Male	Canada	North America	English	Yes	No	No	25-50%	5	2	24
Complete	Male	USA	North America	English	Yes	No	No	25-50%	7	4	5
Complete	Male	USA	North America	English	Yes	Yes	Yes	25-50%	29	30	4
Complete	Male	USA	North America	English	Yes	Yes	Yes	51-75%	5	4	8
Complete	Male	USA	North America	English	Yes	Yes	Yes	<25%	35	13.5	3.5
Complete	Male	USA	North America	English	Yes	No	No	<25%	4	4	10
Incomplete					Yes	Yes	Yes	25-50%	10	4	6
Complete	Female	USA	North America	English	Yes	No	No		0		
Complete	Male	Puerto Rico	North America	English	Yes	Yes	No	<25%	16	1	4
Complete	Male	Canada	North America	English	Yes	Yes	Yes	51-75%	6	7	10
Incomplete					Yes	Yes	Yes	25-50%	19	2	3
Incomplete					Yes	Yes	Yes	<25%	13	10	5
Incomplete					Yes	No	No	<25%	6	2	4
Complete	Male	Canada	North America	English	Yes	Yes	Yes	<25%	41	55	2.5
Complete	Male	USA	North America	English	Yes	No	Yes	25-50%	13	5	4
Complete	Male	USA	North America	English	Yes	No	No	25-50%	12	3	8
Complete	Male	USA	North America	English	Yes	No	No	25-50%	7	1	4
Complete	Male	USA	North America	English	Yes	Yes	Yes	25-50%	17	15	7
Complete	Male	USA	North America	English	Yes	No	Yes	25-50%	10	3	6
Complete	Male	USA	North America	English	Yes	Yes	Yes	<25%	24	14	3
Complete	Female	USA	North America	English	Yes	Yes	No	25-50%	2	1	4
Complete	Male	USA	North America	English	Yes	No	Yes	25-50%	5	6	8
Complete	Female	USA	North America	English	Yes	No	No	25-50%	23	4	8
Complete	Male	Germany	Europe	Non-English	Yes	No	Yes	25-50%	17	20	1
Incomplete					Yes	Yes	Yes	>75%	12		0.5
Complete	Male	USA	North America	English	Yes	Yes	Yes	>75%	25	10	4
Complete	Male	USA	North America	English	Yes	Yes	No	>75%	9	7	6
Complete	Male	USA	North America	English	Yes	Yes	Yes	51-75%	4	2	7
Complete	Male	USA	North America	English	Yes	Yes	Yes	>75%	11	3	7
Complete	Male	Canada	North America	English	Yes	No	Yes	51-75%	10	10	8
Complete	Male	USA	North America	English	Yes	No	No		18	2	40
Complete	Female	Canada	North America	English	Yes	No	No		7		
Complete	Male	USA	North America	English	Yes	No	No	<25%	11	1	6
Complete	Male	USA	North America	English	No	Yes	No		7		
Complete	Male	Belgium	Europe	Non-English	No	No	No		0		

Complete	Male	USA	North America	English	Yes	No	No	51-75%	6	2	12
Complete	Female	Canada	North America	English	Yes	Yes	Yes	25-50%	4	10	12
Complete	Male	USA	North America	English	Yes	No	No	25-50%	9	2	3
Incomplete					Yes	No	Yes	25-50%	16	5	6
Complete	Male	USA	North America	English	Yes	Yes	Yes	25-50%	32	4	2
Complete	Male	USA	North America	English	Yes	Yes	Yes	25-50%	22	13.5	4.5
Incomplete					Yes	Yes	Yes	<25%	9	7.5	6
Complete	Male	USA	North America	English	Yes	Yes	Yes	51-75%	9	8	6
Incomplete					Yes	No	No	25-50%	19	4	15
Incomplete					Yes	Yes	Yes	25-50%	9	8	2
Complete	Female	USA	North America	English	Yes	No	No	25-50%		2	5
Complete	Male	USA	North America	English	Yes	Yes	No		3	3	16
Complete	Male	Spain	Europe	Non-English	Yes	Yes	No		4	6	8
Complete	Male	Australia	Other	English	Yes	No	No	<25%	3	3	4
Complete	Male	USA	North America	English	Yes	No	No	25-50%	7	4	7.5
Complete	Female	USA	North America	English	Yes	No	No	51-75%	5	4	4
Complete	Male	Canada	North America	English	Yes	Yes	Yes	25-50%	10	10	3
Complete	Male	USA	North America	English	Yes	Yes	No	25-50%	5	2	5
Complete	Male	USA	North America	English	Yes	No	Yes	25-50%	11	2	25
Complete	Male	UK	Europe	English	Yes	No	No	51-75%	2	8	6
Complete	Male	Chile	Other	Non-English	Yes	Yes	No	25-50%	14	3	5
Complete	Male	USA	North America	English	No	Yes	No		1		
Complete	Female	USA	North America	English	Yes	No	No		1	1	2
Complete	Male	USA	North America	English	Yes	No	Yes	<25%	3	1	4
Complete	Female	USA	North America	English	Yes	No	No	25-50%	4	3.5	6
Complete	Male	USA	North America	English	Yes	Yes	No	<25%	6	7	5
Incomplete					No	No	No		0		
Complete	Male	USA	North America	English	Yes	Yes	Yes	51-75%	12	40	1
Complete	Male	Argentina	Other	Non-English	Yes	No	No	25-50%	21	0.5	8
Complete	Male	USA	North America	English	Yes	Yes	Yes	25-50%	17	25	5
Complete	Male	UK	Europe	English	Yes	Yes	Yes	<25%	8	5	2
Complete	Male	Canada	North America	English	Yes	Yes	Yes	51-75%	10	20	8
Complete	Male	USA	North America	English	Yes	No	No	25-50%	10	2	4
Complete	Male	USA	North America	English	Yes	Yes	No		10	2	3
Complete	Male	France	Europe	Non-English	Yes	No	No	25-50%	16	1	4
Complete	Male	USA	North America	English	Yes	Yes	No	25-50%	2	2	5
Complete	Male	Switzerland	Europe	Non-English	Yes	No	No	<25%	22	6	4
Complete	Male	USA	North America	English	Yes	Yes	No	>75%	3	2	10
Complete	Female	Canada	North America	English	Yes	No	No	25-50%	7	4	8
Complete	Male	USA	North America	English	Yes	Yes	Yes	25-50%	27	5	4
Complete	Male	USA	North America	English	Yes	Yes	Yes	51-75%	5	3	6
Complete	Male	USA	North America	English	Yes	No	No	25-50%	8	5	8
Complete	Male	USA	North America	English	Yes	Yes	No	<25%	5	1	5
Complete	Male	USA	North America	English	Yes	Yes	Yes	51-75%	1	5	3
Complete	Female	USA	North America	English	Yes	No	No	51-75%	7	3	4

Complete	Female	Spain	Europe	Non-English	Yes	No	No	25-50%	1	2	4
Complete	Male	USA	North America	English	Yes	Yes	Yes	51-75%	32	9	12.5
Complete	Female	USA	North America	English	Yes	Yes	Yes	<25%		2	10
Complete	Male	USA	North America	English	Yes	Yes	Yes	25-50%	7	10	4
Complete	Female	USA	North America	English	Yes	Yes	Yes	51-75%	30	7.5	6
Incomplete					Yes	Yes	Yes	<25%	6	5	4
Complete	Female	USA	North America	English	Yes	Yes	Yes	25-50%	9	4	2
Complete	Male	USA	North America	English	Yes	Yes	Yes	51-75%	13	10	2
Complete	Male	USA	North America	English	Yes	Yes	No	>75%	3	3	10
Complete	Female	USA	North America	English	Yes	No	No	25-50%	8	1	6
Complete	Male	USA	North America	English	Yes	Yes	Yes	51-75%	7	10	6
Complete	Male	USA	North America	English	Yes	Yes	Yes	51-75%	24	15	1.5
Complete	Male	Switzerland	Europe	Non-English	Yes	Yes	Yes	<25%	9	3	72
Incomplete					Yes	Yes	Yes		13	1	72
Complete	Male	Italy	Europe	Non-English	No	No	No		0		
Complete	Female	USA	North America	English	Yes	Yes	No	<25%	5	6	2
Complete	Female	USA	North America	English	Yes	No	No	25-50%	5	2	6
Complete	Male	USA	North America	English	Yes	Yes	No		8	1	5
Complete	Male	USA	North America	English	No	No	No		3		
Complete	Male	USA	North America	English	Yes	No	No		1	1	5
Complete	Female	USA	North America	English	Yes	Yes	Yes	<25%		4	2
Complete	Male	USA	North America	English	Yes	Yes	Yes	25-50%	12	5	1
Complete	Male	Canada	North America	English	Yes	Yes	No	25-50%	9	10	9
Complete	Female	USA	North America	English	Yes	Yes	Yes	>75%	6	7	12
Complete	Male	USA	North America	English	Yes	Yes	Yes	25-50%	15	5	2
Incomplete					Yes	Yes	Yes	25-50%	8	8	8
Complete	Male	USA	North America	English	Yes	Yes	Yes	51-75%	27	50	1
Complete	Male	USA	North America	English	Yes	Yes	Yes	51-75%	25	25	4
Complete	Female	USA	North America	English	Yes	Yes	Yes	<25%	10	20	2.5
Complete	Male	Canada	North America	English	Yes	Yes	Yes	51-75%	5	13.5	2.5
Complete	Male	USA	North America	English	Yes	Yes	No	<25%	1	1	8
Complete	Male	USA	North America	English	Yes	No	Yes	51-75%	10	6	6
Complete	Female	USA	North America	English	No	No	No				
Incomplete					No	No	No		0		
Complete	Male	USA	North America	English	Yes	No	No	25-50%	7	5	10
Complete	Male	USA	North America	English	Yes	No	No	51-75%	11	7.5	5
Complete	Male	USA	North America	English	Yes	No	No		13	1	3
Complete	Female	USA	North America	English	Yes	No	Yes	<25%	11		4
Complete	Male	USA	North America	English	Yes	Yes	No	25-50%	7	1	8
Complete	Male	Columbia	Other	Non-English	Yes	No	No	>75%	5	1	30
Complete	Male	Germany	Europe	Non-English	Yes	Yes	No	25-50%	6	2	8
Complete	Male	USA	North America	English	Yes	Yes	Yes	>75%	12	12.5	8
Incomplete					Yes	No	No	51-75%	4	12	6
Complete	Female	USA	North America	English	Yes	Yes	No	<25%	8	5	3
Complete	Male	USA	North America	English	Yes	Yes	Yes	51-75%	14	50	70

Complete	Male	Canada	North America	English	Yes	Yes	No	<25%	6	3	10
Incomplete					Yes	No	No	<25%	1	1	
Complete	Male	USA	North America	English	Yes	No	No	25-50%		2	4
Complete	Male	Canada	North America	English	Yes	No	Yes		7	5	10
Incomplete					Yes	No	No		11	5	6
Incomplete	Male	USA	North America	English	Yes	No	No	51-75%	1	4	5
Complete	Male	Canada	North America	English	Yes	Yes	Yes	25-50%	22	30	4
Complete	Male	USA	North America	English	Yes	No	No	<25%	2	1	2
Complete	Male	USA	North America	English	Yes	Yes	Yes	25-50%	13	5	2
Complete	Female	Portugal	Europe	Non-English	No	No	No		2		
Complete	Male	USA	North America	English	Yes	Yes	Yes	>75%	16	3	4
Complete	Female	USA	North America	English	Yes	Yes	Yes	25-50%	20	6	5
Complete	Female	USA	North America	English	Yes	Yes	Yes	25-50%	6	3	6
Complete	Female	UK	Europe	English	No	No	No				
Complete	Female	USA	North America	English	Yes	Yes	Yes	25-50%	11	10	6
Complete	Female	USA	North America	English	Yes	Yes	Yes	51-75%	14	6	4
Incomplete					Yes	Yes	Yes	25-50%	19	4	8
Complete	Male	Panama	North America	Non-English	Yes	Yes	Yes		27	20	12
Complete	Male	USA	North America	English	Yes	Yes	Yes	25-50%	15	25	4
Complete	Male	USA	North America	English	Yes	No	No	25-50%	12	2	6
Complete	Male	USA	North America	English	Yes	Yes	Yes	25-50%	28	20	4
Complete	Male	USA	North America	English	Yes	Yes	Yes	25-50%	8	5	10
Complete	Female	Argentina	Other	Non-English	No	No	No		6		
Complete	Male	USA	North America	English	Yes	Yes	Yes	<25%	9	10	5
Complete	Male	Argentina	Other	Non-English	Yes	Yes	Yes	<25%	10	15	6
Complete	Male	Netherlands	Europe	Non-English	Yes	Yes	Yes	51-75%	12	15	3
Complete	Male	Canada	North America	English	Yes	Yes	No	>75%	5	1.5	3
Incomplete					Yes	Yes	Yes	25-50%	6	7.5	4
Complete	Male	USA	North America	English	Yes	Yes	Yes	>75%	8	2	20
Incomplete					Yes	No	No	<25%	8	6	4
Complete	Female	Puerto Rico	North America	English	Yes	No	No		3		
Complete	Male	Canada	North America	English	Yes	Yes	No		5	1.5	6
Complete	Male	USA	North America	English	Yes	Yes	Yes	51-75%	5	5	5
Complete	Female	USA	North America	English	No	Yes	No		10		
Complete	Male	Canada	North America	English	Yes	No	No		4	2	4
Complete	Male	USA	North America	English	Yes	Yes	Yes	>75%	18	10	10
Complete	Female	USA	North America	English	Yes	Yes	No		1	2	5
Complete	Male	USA	North America	English	Yes	No	Yes	25-50%	12	5	4
Complete	Male	USA	North America	English	Yes	Yes	Yes	>75%	18	10	6
Complete	Female	UK	Europe	English	Yes	No	No	51-75%	1	3	8
Incomplete					Yes	Yes	Yes	51-75%	17	6	2
Complete	Male	USA	North America	English	Yes	Yes	Yes	51-75%	10	12	6
Complete	Male	USA	North America	English	Yes	Yes	Yes	<25%	28	15	8
Complete	Female	France	Europe	Non-English	Yes	Yes	No	51-75%	2	3	9
Complete	Female	USA	North America	English	Yes	Yes	Yes	25-50%	18	3	3

Complete	Male	USA	North America	English	Yes	Yes	Yes	25-50%	13	5	5
Incomplete					No	No	No		0		
Complete	Male	USA	North America	English	No	No	No		2		
Complete	Male	USA	North America	English	Yes	Yes	Yes	25-50%	27	15	3
Complete	Male	USA	North America	English	No	No	No		4		
Complete	Female	Argentina	Other	Non-English	No	No	No		3		
Incomplete					Yes	No	No	25-50%	11	10	6
Complete	Male	USA	North America	English	Yes	No	Yes	51-75%	14	5	4
Complete	Male	USA	North America	English	Yes	Yes	Yes	25-50%	2	4	5
Complete	Male	Canada	North America	English	Yes	Yes	Yes	<25%	16	10	10
Complete	Male	USA	North America	English	Yes	Yes	Yes	25-50%	22	5	3
Complete	Male	USA	North America	English	Yes	Yes	Yes	51-75%	8	7	4
Complete	Female	Mexico	North America	Non-English	Yes	No	No	<25%	15	10	72
Complete	Male	USA	North America	English	Yes	No	No	<25%	28	8	4
Complete	Female	Canada	North America	English	No	No	No		2		
Complete	Male	USA	North America	English	Yes	Yes	Yes	25-50%	16	8	3
Complete	Male	USA	North America	English	Yes	Yes	No	25-50%	19	7.5	3
Complete	Female	USA	North America	English	Yes	Yes	No	25-50%	1	2	6
Complete	Female	USA	North America	English	Yes	Yes	No	<25%	5	5	3
Complete	Male	Canada	North America	English	Yes	No	No	25-50%	6	4	10
Incomplete					Yes	No	No	<25%	3	1	4
Complete	Female	USA	North America	English	Yes	No	No		4	1	6
Complete	Female	USA	North America	English	Yes	Yes	No	51-75%	8	1	5
Complete	Female	USA	North America	English	Yes	No	No	<25%	2	18	3
Complete	Male	USA	North America	English	No	No	No				
Complete	Male	Spain	Europe	Non-English	Yes	No	No	51-75%	6	2	24
Complete	Male	Brazil	Other	Non-English	Yes	No	Yes	51-75%	19	5	2
Complete	Female	USA	North America	English	Yes	Yes	Yes	51-75%	16	10	6
Complete	Male	USA	North America	English	Yes	No	No	51-75%	14	2	4
Complete	Male	USA	North America	English	Yes	Yes	Yes	25-50%	13	10	2
Complete	Female	Canada	North America	English	Yes	Yes	Yes	25-50%	6	5	12
Complete	Male	USA	North America	English	Yes	No	No	51-75%	14	1	10
Complete	Female	USA	North America	English	No	Yes	No		4		
Complete	Male	USA	North America	English	Yes	Yes	Yes	25-50%	27	12	3
Complete	Male	Canada	North America	English	No	No	No		5		
Complete	Female	Canada	North America	English	Yes	No	No	25-50%	6	1	1
Complete	Male	Panama	North America	Non-English	Yes	Yes	Yes	>75%	6	10	6
Complete	Male	USA	North America	English	Yes	Yes	Yes	25-50%	5	6	6
Complete	Male	USA	North America	English	Yes	Yes	Yes	51-75%	8	12	3
Complete	Male	USA	North America	English	Yes	No	Yes	25-50%	9	7	8
Complete	Female	USA	North America	English	Yes	No	No	51-75%	9	3	24
Complete	Male	USA	North America	English	No	No	No				
Complete	Female	Venezuela	Other	Non-English	Yes	No	No	25-50%	5	2	4
Complete	Male	UK	Europe	English	Yes	Yes	Yes	51-75%	10	30	45
Complete	Male	Canada	North America	English	Yes	No	Yes	25-50%	17	10	2

Incomplete					Yes	Yes	Yes	25-50%	8	20	5
Complete	Male	USA	North America	English	Yes	No	Yes	25-50%	17	20	3
Complete	Male	Canada	North America	English	Yes	Yes	No		6	1	10
Complete	Male	USA	North America	English	Yes	Yes	Yes	25-50%	16	2	4
Complete	Male	USA	North America	English	Yes	Yes	Yes	>75%	6	2	4
Complete	Female	USA	North America	English	Yes	Yes	Yes	51-75%	20	8	5
Complete	Male	USA	North America	English	Yes	Yes	Yes	>75%	7	5	8
Complete	Male	USA	North America	English	Yes	No	Yes	25-50%	9	5	10
Incomplete	Female	USA	North America	English	No	Yes	No		1		
Complete	Male	Canada	North America	English	Yes	Yes	Yes	51-75%	9	8	3
Complete	Male	USA	North America	English	No	No	No		1		
Complete	Female	USA	North America	English	Yes	No	No	<25%	4	2	10
Complete	Male	USA	North America	English	Yes	No	Yes	25-50%		10	10
Complete	Male	Germany	Europe	Non-English	Yes	Yes	Yes	25-50%	15	50	2
Incomplete					No	Yes	No		2		
Complete	Male	France	Europe	Non-English	Yes	Yes	No	>75%	9	1	8
Incomplete					No	No	No		6		
Complete	Male	USA	North America	English	Yes	Yes	Yes	51-75%	19	24	8
Complete	Male	USA	North America	English	Yes	Yes	Yes	25-50%	16	3	3
Complete	Male	USA	North America	English	Yes	Yes	Yes	51-75%	14	30	4
Complete	Male	Switzerland	Europe	Non-English	Yes	No	No		4	1	10
Complete	Male	Japan	Other	Non-English	Yes	Yes	Yes	25-50%	13	10	12
Complete	Male	Finland	Europe	Non-English	Yes	Yes	No	>75%	7	10	6
Complete	Female	Canada	North America	English	Yes	Yes	Yes	25-50%	20	10	5
Complete	Male	USA	North America	English	Yes	Yes	Yes	<25%	40	15	2
Complete	Male	USA	North America	English	Yes	Yes	Yes	<25%	13	1	20
Complete	Male	USA	North America	English	Yes	No	Yes	25-50%	19		
Complete	Female	USA	North America	English	Yes	Yes	Yes	25-50%	10	12	4
Complete	Male	Mexico	North America	Non-English	No	No	No		8		
Complete	Male	USA	North America	English	Yes	Yes	Yes	<25%	23	2	4
Complete	Male	Brazil	Other	Non-English	Yes	No	No	<25%	8	2	10
Complete	Female	USA	North America	English	Yes	Yes	Yes	51-75%	13	10	2
Complete	Male	USA	North America	English	Yes	No	No	>75%	6	1	3
Complete	Male	USA	North America	English	Yes	Yes	Yes	25-50%	17	2	12
Complete	Male	USA	North America	English	Yes	Yes	Yes	25-50%	28	30	8
Complete	Female	USA	North America	English	Yes	Yes	Yes	25-50%	14	12	5
Complete	Female	USA	North America	English	Yes	Yes	Yes	51-75%	6	5	4.5
Complete	Male	USA	North America	English	No	No	No		6		
Complete	Female	USA	North America	English	Yes	Yes	Yes	<25%	8	1	10
Complete	Female	USA	North America	English	Yes	No	No			2	7
Complete	Female	USA	North America	English	Yes	Yes	Yes	25-50%	19	5	8
Complete	Male	USA	North America	English	Yes	No	No	<25%	8	5	4
Complete	Female	USA	North America	English	Yes	Yes	Yes	25-50%	1	1	5
Complete	Female	Germany	Europe	Non-English	Yes	No	Yes	51-75%	15	10	6
Complete	Male	USA	North America	English	Yes	Yes	No	>75%	3	2	6

Incomplete					No	No	No				
Complete	Male	Canada	North America	English	Yes	Yes	Yes	51-75%	10	20	3
Incomplete					Yes	No	No	<25%	6	1	5
Complete	Male	UK	Europe	English	Yes	Yes	Yes	25-50%	10	20	4
Complete	Male	Canada	North America	English	Yes	No	No	25-50%	6	5	4
Complete	Male	USA	North America	English	Yes	Yes	Yes	25-50%	10	10	6
Complete	Male	Norway	Europe	Non-English	Yes	Yes	Yes	51-75%	18	6	8
Complete	Male	USA	North America	English	Yes	Yes	Yes	<25%	14	5	5
Complete	Male	USA	North America	English	Yes	No	Yes	<25%	1	4	6
Complete	Female	Mexico	North America	Non-English	Yes	No	Yes	>75%	4	2	3
Complete	Male	USA	North America	English	Yes	Yes	No	25-50%	10	1	5
Complete	Male	USA	North America	English	Yes	Yes	Yes	51-75%	11	3	12
Complete	Female	Canada	North America	English	Yes	Yes	No	51-75%	2	10	6
Complete	Male	Germany	Europe	Non-English	Yes	Yes	Yes	51-75%	14	20	8
Complete	Male	USA	North America	English	Yes	Yes	Yes		15	1.5	10
Complete	Male	USA	North America	English	Yes	Yes	Yes	51-75%	20	20	8
Incomplete					Yes	No	No	51-75%	17	7.5	3
Complete	Female	Canada	North America	English	Yes	No	No	25-50%	9	6	6
Incomplete					Yes	No	Yes	51-75%	4	1	2
Complete	Male	USA	North America	English	Yes	No	No	51-75%	8	4	8
Complete	Male	USA	North America	English	Yes	Yes	No		4	2.5	6.5
Complete	Male	USA	North America	English	Yes	Yes	Yes	>75%	14	3	30
Complete	Female	USA	North America	English	Yes	No	No	<25%	8	1	2
Complete	Male	USA	North America	English	Yes	Yes	Yes	<25%	4	4	7
Complete	Female	USA	North America	English	Yes	No	No	25-50%	10	3	8
Incomplete					Yes	Yes	Yes	<25%	23	6	6
Complete	Female	USA	North America	English	Yes	No	No	25-50%	5	6	8
Complete	Male	USA	North America	English	Yes	Yes	Yes	25-50%	7	12	4
Complete	Male	Canada	North America	English	Yes	Yes	Yes	25-50%	2	6	5
Complete	Male	USA	North America	English	Yes	Yes	Yes	>75%	15	10	25
Complete	Male	Denmark	Europe	Non-English	Yes	Yes	Yes	51-75%	13	40	5
Incomplete					Yes	Yes	Yes	51-75%	6	5	6
Complete	Male	USA	North America	English	Yes	No	No	25-50%	3	2.5	4
Complete	Male	Germany	Europe	Non-English	Yes	No	No	25-50%	5	5	6
Complete	Male	USA	North America	English	Yes	No	No		2	1	4
Complete	Male	USA	North America	English	Yes	Yes	Yes	<25%	8	5	3
Complete	Female	USA	North America	English	Yes	No	Yes	51-75%	3	1	6
Complete	Male	Argentina	Other	Non-English	Yes	Yes	Yes	25-50%	22	1.5	15
Complete	Male	USA	North America	English	Yes	Yes	No	<25%	12	60	2
Complete	Male	Sweden	Europe	Non-English	Yes	No	Yes	<25%	8	1	5
Incomplete					Yes	Yes	No	<25%	2	1.5	3
Complete	Female	USA	North America	English	Yes	No	Yes	25-50%	8	3	3
Complete	Male	USA	North America	English	Yes	No	Yes	<25%	6	6	6
Complete	Female	USA	North America	English	Yes	Yes	Yes	25-50%	7	2	6
Complete	Female	USA	North America	English	Yes	Yes	Yes	51-75%	8	10	5

Complete	Female	USA	North America	English	Yes	Yes	No	25-50%	7	12	24
Complete	Male	USA	North America	English	Yes	Yes	Yes	51-75%	23	5	4
Complete	Female	USA	North America	English	Yes	Yes	Yes	25-50%	4	15	4
Complete	Male	USA	North America	English	Yes	Yes	Yes	51-75%	16	10	3
Complete	Female	USA	North America	English	Yes	Yes	Yes	<25%	14	5	6
Complete	Female	UK	Europe	English	Yes	No	No	<25%	14	6	2
Complete	Female	USA	North America	English	No	No	No		3		
Complete	Female	USA	North America	English	Yes	No	No	51-75%	5	3	16
Incomplete					Yes	Yes	Yes	25-50%	20	3	5
Complete	Female	USA	North America	English	Yes	Yes	Yes	51-75%	24	16	3
Incomplete					Yes	Yes	Yes	25-50%	10	6	2.5
Complete	Female	France	Europe	Non-English	Yes	Yes	Yes	>75%	9	7	15
Complete	Male	Canada	North America	English	Yes	Yes	Yes	51-75%	3	7	4
Complete	Female	USA	North America	English	Yes	No	No			1	4
Complete	Male	Sweden	Europe	Non-English	Yes	Yes	Yes	51-75%	14	17.5	1.5
Complete	Male	Germany	Europe	Non-English	Yes	Yes	Yes	25-50%	16	25	4
Complete	Male	Italy	Europe	Non-English	Yes	Yes	Yes	51-75%	11	5	24
Complete	Male	USA	North America	English	Yes	Yes	No	<25%	8	3	10
Complete	Female	USA	North America	English	No	No	No		4		
Complete	Male	USA	North America	English	Yes	Yes	Yes	25-50%	24	15	6
Complete	Female	USA	North America	English	Yes	Yes	No	25-50%	20	2	2
Complete	Male	USA	North America	English	No	No	No		3		
Complete	Male	USA	North America	English	Yes	No	No		6	2	4
Complete	Male	Austria	Europe	Non-English	Yes	Yes	No	<25%	6	5	5
Complete	Male	USA	North America	English	Yes	Yes	No	25-50%	9	5	6
Complete	Female	USA	North America	English	Yes	No	No	25-50%	6	2	12
Complete	Male	USA	North America	English	Yes	Yes	Yes	25-50%	19	15	6
Complete	Female	Canada	North America	English	Yes	Yes	Yes	25-50%	6	1	8
Complete	Male	Brazil	Other	Non-English	Yes	Yes	Yes	25-50%	10	1	20
Complete	Female	USA	North America	English	Yes	Yes	No	25-50%	10	3	6
Complete	Male	USA	North America	English	Yes	Yes	Yes	51-75%	8	6	5
Complete	Male	USA	North America	English	Yes	Yes	Yes	51-75%	9	8	3
Complete	Male	Canada	North America	English	Yes	Yes	Yes	25-50%	28	15	3
Complete	Male	USA	North America	English	Yes	Yes	Yes	<25%	31	6	2
Complete	Male	USA	North America	English	Yes	Yes	No	25-50%	3	3	4
Complete	Male	Australia	Other	English	Yes	Yes	Yes	25-50%	14	25	5
Complete	Male	USA	North America	English	Yes	Yes	Yes	51-75%	5	5	2
Complete	Male	Chile	Other	Non-English	Yes	No	No	25-50%	4	4	10
Complete	Male	Mexico	North America	Non-English	Yes	Yes	No	25-50%	18	12	4
Complete	Male	USA	North America	English	Yes	Yes	Yes	25-50%	10	7	8
Complete	Female	USA	North America	English	Yes	No	No		9	1	12
Complete	Male	Spain	Europe	Non-English	Yes	Yes	Yes	25-50%	2	5	4
Complete	Male	USA	North America	English	Yes	Yes	Yes	25-50%	17	4	8
Complete	Male	USA	North America	English	Yes	No	No	<25%	6	4	4
Complete	Male	Canada	North America	English	Yes	No	Yes	<25%	8	3	6

Complete	Male	Germany	Europe	Non-English	Yes	No	No	51-75%	10	4	16
Complete	Female	USA	North America	English	Yes	Yes	Yes	<25%	9	10	3
Complete	Male	USA	North America	English	Yes	No	No	>75%	8	6	8
Complete	Female	Australia	Other	English	Yes	No	No	<25%	1	2	6
Complete	Male	USA	North America	English	Yes	Yes	Yes	51-75%	7	15	3
Complete	Male	USA	North America	English	Yes	No	Yes	25-50%	3	5	8
Complete	Female	Canada	North America	English	Yes	Yes	Yes	25-50%	4	1	6
Complete	Male	USA	North America	English	Yes	Yes	Yes	>75%	5	10	8
Complete	Male	Canada	North America	English	Yes	No	Yes	<25%	8	4	8
Incomplete					No	No	No		1		
Complete	Male	France	Europe	Non-English	Yes	Yes	Yes	25-50%	12	15	4
Complete	Male	USA	North America	English	Yes	No	No	25-50%	5	8	1.5
Complete	Female	USA	North America	English	No	No	No		29		
Complete	Male	USA	North America	English	Yes	No	No	25-50%	6	6	6
Incomplete					Yes	No	No	<25%	10	1	20
Incomplete					No	No	No		5		
Complete	Female	USA	North America	English	Yes	Yes	Yes	25-50%	9	10	5
Incomplete					Yes	Yes	No	25-50%	9	12	4
Incomplete					No	No	No				
Complete	Male	USA	North America	English	Yes	No	Yes	25-50%	5	2	6
Complete	Female	USA	North America	English	No	No	No		3		
Complete	Female	Argentina	Other	Non-English	No	No	No		4		
Complete	Male	USA	North America	English	Yes	No	No	<25%	18	10	8
Complete	Female	USA	North America	English	No	No	No		0		
Complete	Male	Canada	North America	English	Yes	No	No	25-50%	1	3	3
Complete	Male	USA	North America	English	Yes	Yes	Yes	<25%	20	12	8
Complete	Male	USA	North America	English	Yes	No	No	<25%	9	6	3
Complete	Female	Canada	North America	English	Yes	No	Yes	25-50%	3	5	4
Complete	Male	Canada	North America	English	Yes	No	No	>75%	6	3	3
Complete	Male	UK	Europe	English	Yes	Yes	Yes	25-50%	12	12	3
Complete	Male	USA	North America	English	Yes	No	No	51-75%	0	4	6
Complete	Male	USA	North America	English	Yes	Yes	No	25-50%	5	10	40
Incomplete					Yes	Yes	Yes	51-75%	1	3	10
Complete	Male	Australia	Other	English	Yes	Yes	Yes	>75%	8	50	4
Complete	Female	Australia	Other	English	Yes	Yes	Yes	51-75%	7	30	4
Complete	Male	France	Europe	Non-English	Yes	No	No	51-75%	5	1.5	6
Complete	Male	Canada	North America	English	Yes	No	No	<25%	10	5	10
Complete	Male	USA	North America	English	Yes	No	Yes	>75%		1	40
Complete	Male	UK	Europe	English	No	Yes	No		7		
Complete	Male	USA	North America	English	Yes	Yes	Yes	25-50%	7	5	3
Complete	Female	USA	North America	English	Yes	No	No	25-50%	11	8	4
Incomplete					Yes	No	No			1	3
Complete	Male	UK	Europe	English	Yes	Yes	Yes	25-50%	17	6	3
Complete	Male	UK	Europe	English	Yes	No	No		4	1	4
Complete	Female	Poland	Europe	Non-English	Yes	No	No	25-50%	4	4	6

Complete	Male	Germany	Europe	Non-English	Yes	Yes	Yes	51-75%	9	18	14
Complete	Male	USA	North America	English	Yes	Yes	No	>75%	5	5	3
Complete	Female	Germany	Europe	Non-English	Yes	No	No	25-50%	3	2	40
Complete	Male	USA	North America	English	Yes	No	No	51-75%	3	6	8
Complete	Male	Australia	Other	English	Yes	Yes	Yes	51-75%	15	9	8
Complete	Male	USA	North America	English	Yes	No	No	25-50%	3	3	4
Complete	Male	USA	North America	English	Yes	Yes	Yes	>75%	5	20	5
Complete	Male	Venezuela	Other	Non-English	Yes	Yes	No		1	2	15
Complete	Male	USA	North America	English	Yes	Yes	Yes	<25%	12	5	5
Incomplete	Female				Yes	Yes	No	<25%			
Complete	Male	India	Other	Non-English	Yes	No	Yes	>75%	43	10	6
Incomplete					Yes	No	Yes	51-75%	6	20	5
Complete	Male	Switzerland	Europe	Non-English	No	No	No				
Complete	Female	USA	North America	English	Yes	No	No	<25%	20		5
Complete	Male	USA	North America	English	Yes	Yes	No	>75%	3	5	9
Incomplete					Yes	Yes	No	<25%	8	1	5
Complete	Male	Switzerland	Europe	Non-English	Yes	No	No			2	10
Incomplete					Yes	Yes	No	25-50%	7	7	5
Complete	Male	USA	North America	English	Yes	Yes	No	25-50%	6	3	3
Complete	Male	Canada	North America	English	Yes	No	No	25-50%	8	4	8
Complete	Male	USA	North America	English	Yes	Yes	No	>75%	6	1.5	8
Complete	Female	Switzerland	Europe	Non-English	Yes	Yes	Yes		8	2	6
Complete	Female	USA	North America	English	Yes	Yes	Yes	<25%	8	4	6
Complete	Male	USA	North America	English	Yes	No	Yes	>75%	2	3	10
Complete	Female	USA	North America	English	Yes	Yes	No	51-75%	6	7.5	5
Complete	Male	USA	North America	English	Yes	Yes	Yes	25-50%	27	30	4
Complete	Male	USA	North America	English	Yes	No	Yes	25-50%	3	4	4
Complete	Male	USA	North America	English	Yes	No	Yes	<25%	28	4	4
Incomplete					No	Yes	No		0		
Complete	Male	Netherlands	Europe	Non-English	Yes	No	No	25-50%	6	1	5
Complete	Male	Barbados	North America	English	Yes	No	No	<25%	35	3	4
Complete	Male	Denmark	Europe	Non-English	No	No	No		1		
Complete	Male	USA	North America	English	Yes	No	No	51-75%	5	3	6
Complete	Female	UK	Europe	English	Yes	No	No	25-50%	3	1	5
Complete	Male	USA	North America	English	Yes	Yes	Yes	<25%	22	2	8
Complete	Female	USA	North America	English	Yes	Yes	No	25-50%	4	8	4
Complete	Female	USA	North America	English	Yes	Yes	No	25-50%	6	3	12
Incomplete					Yes	Yes	Yes	25-50%	28	20	4
Complete	Female	USA	North America	English	Yes	Yes	Yes	51-75%	6	3	12
Complete	Male	Netherlands	Europe	Non-English	Yes	No	No	25-50%	21	4	4
Incomplete					Yes	No	No	51-75%	7	3	3
Complete	Male	USA	North America	English	Yes	Yes	Yes	51-75%	15	6	4
Incomplete					Yes	Yes	Yes	>75%	10	18	2
Complete	Male	USA	North America	English	Yes	No	No	25-50%	15	4	3
Incomplete					Yes	Yes	No		13	6	6

Complete	Female	USA	North America	English	Yes	No	No	<25%	10	25	3
Complete					Yes	No	No	51-75%	5	1	2
Complete	Male	USA	North America	English	Yes	Yes	Yes	>75%	6	35	3
Complete	Male	Canada	North America	English	Yes	Yes	Yes	51-75%	5	15	6
Complete	Female	USA	North America	English	Yes	Yes	Yes	<25%	10	10	5
Complete	Male	USA	North America	English	No	No	No				
Complete	Male	Australia	Other	English	Yes	No	No	<25%	4	1	4
Incomplete	Male	USA	North America	English	Yes	Yes	Yes	51-75%	8	6	30
Complete	Male	Netherlands	Europe	Non-English	Yes	No	No	<25%	1	3	8
Complete	Male	Netherlands	Europe	Non-English	Yes	Yes	Yes	51-75%	28	26	2
Complete	Male	Switzerland	Europe	Non-English	Yes	Yes	Yes	51-75%	10	20	4
Complete	Female	Germany	Europe	Non-English	Yes	No	No	25-50%	10	4	8
Complete	Male	UK	Europe	English	Yes	No	No	51-75%	4	7	4
Complete	Female	Switzerland	Europe	Non-English	Yes	Yes	Yes	<25%	13	13.5	4
Complete	Male	UK	Europe	English	Yes	Yes	Yes	<25%	10	3	4
Complete	Male	Germany	Europe	Non-English	Yes	Yes	Yes	51-75%	23	40	3
Complete	Male	Netherlands	Europe	Non-English	Yes	No	No	25-50%	6	4	2
Complete	Male	Germany	Europe	Non-English	No	No	No		0		
Complete	Male	Finland	Europe	Non-English	Yes	Yes	Yes	<25%	14	7.5	15
Complete	Female	Belgium	Europe	Non-English	Yes	Yes	Yes	51-75%	5	16	12
Complete	Male	UK	Europe	English	Yes	Yes	Yes	51-75%	10	12	3
Complete	Female	USA	North America	English	Yes	No	No	<25%	8	2	5
Complete	Male	Netherlands	Europe	Non-English	Yes	No	Yes	25-50%	9	5	6
Complete	Male	Spain	Europe	Non-English	Yes	No	No	>75%	10	1	10
Complete	Male	Finland	Europe	Non-English	Yes	Yes	Yes	51-75%	6	10	5
Complete	Male	Norway	Europe	Non-English	Yes	Yes	Yes	51-75%	12	15	8
Complete	Female	UK	Europe	English	Yes	No	No	>75%	8	5	15
Complete	Male	USA	North America	English	Yes	Yes	Yes	25-50%	1	5	4
Complete	Female	USA	North America	English	Yes	Yes	Yes	>75%	6	4	7
Complete	Male	Netherlands	Europe	Non-English	Yes	Yes	Yes	25-50%	16	12	2
Complete	Male	USA	North America	English	Yes	No	No	51-75%	2	2	8
Complete	Female	Australia	Other	English	Yes	Yes	Yes	51-75%	0	3	6
Complete	Female	Sweden	Europe	Non-English	Yes	Yes	Yes	25-50%	6	5	4
Complete	Female	UK	Europe	English	Yes	Yes	No	51-75%	3	1.5	8
Complete	Male	USA	North America	English	Yes	No	No	25-50%	5	1	6
Complete	Male	UK	Europe	English	Yes	Yes	No	<25%	10	12	10
Complete	Female	USA	North America	English	Yes	Yes	Yes	51-75%	25	8	4
Complete	Female	Switzerland	Europe	Non-English	Yes	Yes	Yes	25-50%	10	8	8
Complete	Male	USA	North America	English	Yes	Yes	Yes	25-50%	15	3	3
Complete	Male	USA	North America	English	Yes	No	Yes	51-75%	28	1	3
Complete	Female	USA	North America	English	Yes	Yes	No	25-50%	7	2	4
Complete	Male	USA	North America	English	Yes	No	No	<25%	23	5	3
Complete	Male	Netherlands	Europe	Non-English	Yes	Yes	No	<25%	12	2	8
Complete	Male	USA	North America	English	Yes	Yes	Yes	51-75%	4	10	6
Complete	Male	USA	North America	English	Yes	No	No	25-50%	35	20	10

Complete	Male	USA	North America	English	Yes	Yes	Yes	25-50%	11	2	20
Complete	Male	Canada	North America	English	Yes	Yes	Yes	25-50%	7	12	6
Complete	Male	France	Europe	Non-English	Yes	Yes	Yes	51-75%	8	10	3
Complete	Male	Canada	North America	English	Yes	Yes	No	51-75%	9	5	2
Complete	Male	USA	North America	English	Yes	Yes	Yes	>75%	23	20	3
Complete	Male	Canada	North America	English	Yes	No	No	<25%	30	25	2
Complete	Female	Sweden	Europe	Non-English	Yes	No	No	51-75%	6	5	32
Complete	Male	Canada	North America	English	Yes	Yes	No	<25%	8	4	3
Complete	Male	USA	North America	English	Yes	No	No	25-50%	3	4	12
Complete	Male	USA	North America	English	Yes	Yes	Yes	25-50%	22	1	6
Complete	Female	Canada	North America	English	Yes	Yes	Yes	25-50%	7	18	2
Complete	Female	Canada	North America	English	Yes	No	Yes	25-50%	23	5	9
Complete	Female	USA	North America	English	Yes	No	No	<25%	8	3	8
Complete	Male	USA	North America	English	Yes	Yes	Yes	25-50%	9	10	5
Complete	Female	New Zealand	Other	English	Yes	No	No	<25%	10	1	2
Complete	Male	USA	North America	English	Yes	No	Yes	25-50%	9	2.5	8
Complete	Male	USA	North America	English	Yes	No	No	25-50%	3	3	8
Complete	Female	USA	North America	English	Yes	Yes	Yes	>75%	2	3	4
Complete	Male	USA	North America	English	Yes	Yes	Yes	25-50%	15	25	2
Complete	Male	Italy	Europe	Non-English	Yes	Yes	No	51-75%	5	3	8
Complete	Female	Germany	Europe	Non-English	Yes	Yes	Yes		16	20	5
Complete	Male	UK	Europe	English	Yes	Yes	Yes	51-75%	13	6	2
Complete	Female	Ireland	Europe	English	Yes	Yes	No	51-75%	6	3	7
Complete	Female	France	Europe	Non-English	Yes	Yes	Yes	<25%	30	10	7
Complete	Male	Sweden	Europe	Non-English	Yes	Yes	Yes	25-50%	10	15	4
Complete	Female	Ireland	Europe	English	Yes	Yes	No		2	1	20
Complete	Male	Netherlands	Europe	Non-English	Yes	No	No	<25%	6	5	20
Complete	Male	France	Europe	Non-English	Yes	Yes	Yes	51-75%	11	20	3
Complete	Female	Germany	Europe	Non-English	No	No	No		1		
Complete	Female	France	Europe	Non-English	Yes	No	No	51-75%	6	4	15
Complete	Male	Canada	North America	English	Yes	Yes	Yes	>75%	3	3	8
Complete	Male	France	Europe	Non-English	Yes	No	No	51-75%	14	3	12
Complete	Male	Spain	Europe	Non-English	Yes	Yes	No	<25%	3	5	8
Complete	Male	France	Europe	Non-English	Yes	Yes	Yes	>75%	6	15	6
Complete	Male	Germany	Europe	Non-English	Yes	Yes	No	25-50%	3	1	7
Complete	Male	France	Europe	Non-English	Yes	Yes	No	51-75%	9	6	6
Complete	Male	USA	North America	English	Yes	Yes	Yes		20	5	5
Complete	Male	France	Europe	Non-English	Yes	Yes	Yes	25-50%	8	7	10
Complete	Male	USA	North America	English	Yes	Yes	Yes	<25%	19	7.5	2.5
Complete	Male	Canada	North America	English	No	No	No		2		
Complete	Male	Italy	Europe	Non-English	Yes	No	No	<25%	5	2	10
Complete	Female	Norway	Europe	Non-English	Yes	No	Yes	25-50%	4	5	12
Complete	Male	USA	North America	English	Yes	Yes	Yes	<25%	22	1	10
Complete	Female	Canada	North America	English	Yes	Yes	Yes	25-50%	17	50	3
Complete	Female	USA	North America	English	No	No	No		0		

Complete	Female	Canada	North America	English	Yes	No	No	25-50%	5	3	8
Complete	Male	USA	North America	English	Yes	Yes	Yes	>75%	5	8	5
Complete	Male	France	Europe	Non-English	Yes	Yes	Yes	51-75%	15	3	6
Complete	Male	France	Europe	Non-English	No	No	No		3		
Complete	Male	France	Europe	Non-English	Yes	Yes	Yes	51-75%	12	15	6
Complete	Male	Sweden	Europe	Non-English	No	No	No		2		
Complete	Male	Finland	Europe	Non-English	Yes	Yes	Yes	51-75%	20	10	12
Complete	Female	Spain	Europe	Non-English	Yes	No	No	<25%	5	1	4
Complete	Male	USA	North America	English	Yes	No	No	25-50%	2	3	5
Complete	Female	UK	Europe	English	Yes	Yes	Yes	25-50%	14	20	3
Complete	Male	USA	North America	English	Yes	Yes	No	25-50%	6	4	3
Complete	Male	USA	North America	English	Yes	No	No	<25%	10	5	6
Complete	Male	Germany	Europe	Non-English	Yes	No	No	25-50%	6	10	5
Complete	Male	France	Europe	Non-English	Yes	No	No		27	10	10
Complete	Female	USA	North America	English	Yes	No	No	<25%	4	6	4
Complete	Female	USA	North America	English	Yes	No	No	51-75%	1	2	8
Complete	Female	USA	North America	English	Yes	Yes	No	25-50%	4	6	5
Complete	Female	USA	North America	English	Yes	No	Yes	25-50%	1	4	4
Complete	Female	Canada	North America	English	Yes	No	No	>75%	0	2	8
Complete	Female	USA	North America	English	Yes	Yes	Yes	<25%	21	12	5
Complete	Female	USA	North America	English	Yes	Yes	Yes	>75%	18	15	4
Complete	Male	Canada	North America	English	Yes	Yes	Yes	25-50%	29	12	4.5
Complete	Female	Australia	Other	English	No	No	No		4		
Complete	Male	France	Europe	Non-English	Yes	No	No	51-75%	8	2	12
Complete	Female	Italy	Europe	Non-English	No	No	No		9		
Complete	Female	Switzerland	Europe	Non-English	No	No	No		5		
Complete	Male	Austria	Europe	Non-English	Yes	No	No	51-75%	11	3	5
Complete	Female	France	Europe	Non-English	Yes	Yes	No	25-50%	15	2	5
Complete	Male	Canada	North America	English	Yes	Yes	Yes	51-75%	30	6	4
Complete	Male	UK	Europe	English	Yes	Yes	Yes	25-50%	4	6	4
Complete	Female	USA	North America	English	Yes	No	Yes	51-75%	18	10	3
Complete	Female	USA	North America	English	Yes	No	No	25-50%	3	3	4
Complete	Male	USA	North America	English	Yes	Yes	Yes	25-50%	8	2.5	12
Complete	Female	USA	North America	English	Yes	Yes	Yes	25-50%	7	3	2
Complete	Male	USA	North America	English	Yes	No	Yes	25-50%	4	2	3
Complete	Male	USA	North America	English	Yes	No	No	25-50%	24	1	5
Complete	Male	USA	North America	English	Yes	Yes	Yes	25-50%	11	5	6
Complete	Male	France	Europe	Non-English	Yes	Yes	No		12	5	4
Complete	Male	Netherlands	Europe	Non-English	Yes	Yes	Yes	25-50%	26	25	4
Complete	Male	USA	North America	English	Yes	No	Yes	25-50%	19	10	3
Complete	Male	USA	North America	English	Yes	Yes	Yes	<25%	6	8	8
Complete	Male	UK	Europe	English	Yes	Yes	Yes	51-75%	6	12	4
Complete	Male	USA	North America	English	Yes	Yes	No		4	1	3
Complete	Female	UK	Europe	English	Yes	No	No	25-50%	8	4	4
Complete	Male	USA	North America	English	Yes	Yes	Yes	25-50%	14	12	8

Complete	Female	Sweden	Europe	Non-English	Yes	No	No		6		
Complete	Female	USA	North America	English	Yes	No	Yes	<25%	10	2.5	3.5
Complete	Female	USA	North America	English	Yes	Yes	No	25-50%	2	6	6
Complete	Female	USA	North America	English	Yes	Yes	No	25-50%	8	1	6
Complete	Male	USA	North America	English	Yes	Yes	Yes	<25%	37	12.5	5
Complete	Male	USA	North America	English	Yes	Yes	Yes	>75%	32	15	3
Complete	Male	Canada	North America	English	Yes	Yes	Yes	51-75%	4	4	10
Complete	Male	USA	North America	English	Yes	Yes	Yes	51-75%	11	1	10
Complete	Female	Germany	Europe	Non-English	Yes	No	No	25-50%	8	3	4
Complete	Male	USA	North America	English	Yes	Yes	Yes	25-50%	1	3	4
Complete	Male	France	Europe	Non-English	Yes	Yes	Yes	<25%	16	5	10
Complete	Male	Ireland	Europe	English	Yes	Yes	Yes	<25%	9	15	4
Complete	Male	Canada	North America	English	Yes	No	No	25-50%	9	5	10
Complete	Male	Finland	Europe	Non-English	Yes	Yes	No		3	1	20
Complete	Male	France	Europe	Non-English	Yes	Yes	Yes	25-50%	9	8	3
Complete	Male	USA	North America	English	Yes	Yes	Yes	51-75%	11	15	8
Complete	Male	USA	North America	English	Yes	Yes	Yes	25-50%	24	8	3
Complete	Female	USA	North America	English	Yes	Yes	Yes	25-50%	26	12.5	12
Complete	Male	USA	North America	English	Yes	Yes	Yes	>75%	15	6	5
Complete	Female	Sweden	Europe	Non-English	Yes	Yes	Yes	25-50%	4	7.5	10
Complete	Male	Canada	North America	English	Yes	No	No	<25%	5	3	8
Complete	Male	USA	North America	English	Yes	No	Yes	<25%	4	4	3
Complete	Male	Canada	North America	English	Yes	Yes	Yes	>75%	15	7	7
Complete	Female	zech Republi	Europe	Non-English	Yes	No	No	25-50%	5	2	6
Complete	Male	UK	Europe	English	Yes	Yes	Yes	<25%	12	10	12
Complete	Female	Sweden	Europe	Non-English	Yes	Yes	No		4	2.5	9
Complete	Female	Canada	North America	English	Yes	Yes	No	51-75%	6	2	4
Complete	Male	USA	North America	English	Yes	Yes	No	<25%	6	2	10
Complete	Male	Finland	Europe	Non-English	Yes	Yes	No	25-50%	7	8	3
Complete	Male	USA	North America	English	Yes	No	Yes	>75%	19	10	60
Complete	Male	USA	North America	English	Yes	No	No	<25%	2	1.5	6
Complete	Female	Canada	North America	English	Yes	No	Yes	25-50%	20	10	2
Complete	Female	USA	North America	English	Yes	Yes	Yes	51-75%	13	3	6
Complete	Male	USA	North America	English	No	No	No		4		
Complete	Female	Germany	Europe	Non-English	Yes	No	Yes	25-50%	23	5	5
Complete	Male	Spain	Europe	Non-English	Yes	No	No	<25%	12	6	4
Complete	Female	USA	North America	English	Yes	Yes	Yes	51-75%	5	4.5	5.5
Complete	Female	USA	North America	English	Yes	No	No	<25%	10	2	7
Complete	Female	UK	Europe	English	Yes	No	Yes	<25%	3	1	8
Complete	Male	USA	North America	English	Yes	Yes	Yes	51-75%	5	8	4
Complete	Female	USA	North America	English	Yes	No	No	<25%	3	2	8
Complete	Male	Brazil	Other	Non-English	Yes	No	No	25-50%	0	3	30
Complete	Female	Canada	North America	English	No	No	No		1		
Complete	Female	Australia	Other	English	Yes	No	Yes	25-50%	6	1	16
Complete	Male	Switzerland	Europe	Non-English	Yes	No	No	<25%	7	10	6

Complete	Male	USA	North America	English	Yes	Yes	Yes	51-75%	4	10	10
Complete	Female	USA	North America	English	No	Yes	No		5		
Complete	Male	Spain	Europe	Non-English	Yes	Yes	Yes	25-50%	10	8	8
Complete	Female	UK	Europe	English	Yes	Yes	Yes	51-75%	10	5	12
Complete	Male	USA	North America	English	Yes	Yes	Yes	25-50%	7	15	5
Complete	Male	Australia	Other	English	Yes	Yes	Yes	25-50%	13	40	2.5
Complete	Male	New Zealand	Other	English	Yes	No	No	25-50%	2	5	3
Complete	Male	Germany	Europe	Non-English	Yes	No	No	<25%	4	5	15
Complete	Male	USA	North America	English	Yes	No	Yes	25-50%	8	5	2
Complete	Male	USA	North America	English	Yes	No	No	<25%	16	1	
Complete	Male	USA	North America	English	No	No	No		1		
Complete	Female	USA	North America	English	Yes	Yes	Yes	25-50%	8	12	4
Complete	Female	France	Europe	Non-English	Yes	No	No	>75%	2	1	12
Complete	Female	Portugal	Europe	Non-English	Yes	No	No	25-50%	18	2	30
Complete	Female	Spain	Europe	Non-English	Yes	Yes	No	<25%	5	2	10
Complete	Male	USA	North America	English	Yes	Yes	Yes	51-75%	5	8	16
Complete	Female	USA	North America	English	No	No	No		0		
Complete	Female	Germany	Europe	Non-English	Yes	Yes	Yes	>75%	12	12	3
Complete	Male	Germany	Europe	Non-English	Yes	Yes	No	25-50%	8	4	3
Complete	Female	Australia	Other	English	Yes	No	No	<25%	7	6	10
Complete	Female	Germany	Europe	Non-English	Yes	Yes	Yes	>75%	11	8	5
Complete	Male	Germany	Europe	Non-English	Yes	Yes	Yes	51-75%	6	10	7
Complete	Male	Germany	Europe	Non-English	Yes	Yes	No	<25%	10	10	6
Complete	Male	Germany	Europe	Non-English	No	No	No				
Complete	Female	Germany	Europe	Non-English	Yes	Yes	Yes	<25%	7	7.5	6
Complete	Female	USA	North America	English	Yes	Yes	Yes	>75%	7	6.5	4
Complete	Female	Germany	Europe	Non-English	Yes	No	No		3	2	10
Complete	Male	Sweden	Europe	Non-English	Yes	Yes	Yes	<25%	1	3	18
Complete	Female	USA	North America	English	Yes	Yes	Yes	25-50%	0	10	3
Complete	Female	USA	North America	English	Yes	Yes	Yes	<25%	19	3	8
Complete	Male	Denmark	Europe	Non-English	No	No	No		3		
Complete	Female	Germany	Europe	Non-English	Yes	No	No	<25%	8	2	6
Complete	Female	Germany	Europe	Non-English	No	No	No		1		
Complete	Female	Germany	Europe	Non-English	Yes	No	No	<25%	8	6	3
Complete	Male	UK	Europe	English	Yes	Yes	Yes	<25%	9	40	3
Complete	Male	Germany	Europe	Non-English	No	No	No		2		
Complete	Female	Canada	North America	English	No	No	No		0		
Complete	Male	Russia	Europe	Non-English	Yes	No	No	<25%	10	2	6
Complete	Male	Germany	Europe	Non-English	Yes	No	No	51-75%	7	1	2
Complete	Male	Germany	Europe	Non-English	Yes	Yes	Yes	25-50%	7	12	6
Complete	Male	USA	North America	English	Yes	Yes	Yes	25-50%	7	12	10
Complete	Male	USA	North America	English	Yes	Yes	Yes	51-75%	14	20	1
Complete	Male	USA	North America	English	Yes	Yes	Yes	25-50%	10	30	2
Complete	Male	Finland	Europe	Non-English	Yes	No	No	51-75%	3	3	10
Complete	Male	USA	North America	English	Yes	Yes	Yes	51-75%	5	5	5

Complete	Male	Canada	North America	English	Yes	No	No	25-50%	6	8	72
Complete	Male	UK	Europe	English	Yes	Yes	No		8	4	12
Complete	Female	USA	North America	English	No	No	No				
Complete	Male	Spain	Europe	Non-English	Yes	Yes	No	<25%	14	4	14
Complete	Female	Canada	North America	English	Yes	No	Yes	25-50%	2	3	15
Complete	Female	Sweden	Europe	Non-English	Yes	No	No	25-50%	7	2	6
Complete	Female	USA	North America	English	No	No	No				
Complete	Female	UK	Europe	English	Yes	Yes	Yes	51-75%	9	12	8
Complete	Male	USA	North America	English	Yes	Yes	Yes	25-50%	30	6	5
Complete	Male	USA	North America	English	Yes	No	Yes	<25%	15	10	3
Complete	Male	Canada	North America	English	Yes	No	No	51-75%	9	3	15
Complete	Male	USA	North America	English	No	Yes	No		3		
Complete	Female	UK	Europe	English	No	Yes	No		5		
Complete	Male	Australia	Other	English	Yes	No	No		4		
Complete	Female	Canada	North America	English	Yes	Yes	Yes	51-75%	12	3.5	20
Complete	Female	Canada	North America	English	Yes	No	No	<25%	4	2	4
Complete	Male	USA	North America	English	Yes	No	Yes	<25%	27	5	2
Complete	Male	USA	North America	English	Yes	No	No	>75%	4	1	8
Complete	Male	USA	North America	English	Yes	Yes	Yes	51-75%	26	12	3
Complete	Male	USA	North America	English	Yes	Yes	Yes	51-75%	41	15	3
Complete	Male	Canada	North America	English	Yes	Yes	No	25-50%	22	6	12
Complete	Male	Canada	North America	English	Yes	Yes	Yes	25-50%	20	7.5	5
Complete	Male	USA	North America	English	Yes	Yes	Yes	51-75%	29	20	6
Complete	Male	USA	North America	English	Yes	No	No	<25%	2	1	6
Complete	Female	USA	North America	English	Yes	No	No		11	2	5
Complete	Male	Netherlands	Europe	Non-English	No	No	No				
Complete	Male	Turkey	Other	Non-English	Yes	No	Yes	<25%	25	3	6
Complete	Female	USA	North America	English	Yes	No	No	<25%	2	1	5
Complete	Male	Canada	North America	English	Yes	Yes	Yes	51-75%	21	30	1.5
Complete	Male	Canada	North America	English	Yes	No	No	<25%	6	3	4
Complete	Male	Canada	North America	English	Yes	Yes	Yes	51-75%	38	15	5
Complete	Male	USA	North America	English	Yes	Yes	Yes	25-50%	10	15	3
Complete	Female	Canada	North America	English	Yes	No	No			1	5
Complete	Male	USA	North America	English	Yes	Yes	Yes	25-50%	5	4	8
Complete	Female	Australia	Other	English	No	Yes	No	<25%	4		
Complete	Female	Canada	North America	English	Yes	No	Yes	25-50%	7	4	4
Complete	Male	Venezuela	Other	Non-English	Yes	No	No	25-50%	30	12	6
Complete	Male	Sweden	Europe	Non-English	Yes	No	No	<25%	18	2	3
Complete	Male	UK	Europe	English	Yes	Yes	Yes	>75%	4	6	5
Complete	Male	France	Europe	Non-English	Yes	Yes	Yes	51-75%	12	15	4
Complete	Male	France	Europe	Non-English	Yes	No	No	25-50%	6	1	6
Complete	Male	France	Europe	Non-English	Yes	No	No	51-75%	5	3	15
Complete	Male	Canada	North America	English	Yes	Yes	Yes	>75%	24	30	10
Complete	Male	France	Europe	Non-English	Yes	No	No		27	5	5
Incomplete	Female				Yes	Yes	Yes				

Complete	Female	Finland	Europe	Non-English	No	Yes	No		2		
Complete	Male	USA	North America	English	Yes	Yes	Yes	>75%	6	8	8
Complete	Male	ted arab emira	Other	Non-English	Yes	No	No		12	1	2
Complete	Male	Canada	North America	English	Yes	No	No	51-75%	10	2	5
Complete	Female	France	Europe	Non-English	Yes	No	No	51-75%	6	7	24
Complete	Male	Sweden	Europe	Non-English	Yes	No	No	51-75%	8	4	4
Incomplete					Yes	Yes	Yes	51-75%	18	3	20
Complete	Female	USA	North America	English	Yes	Yes	Yes	>75%	13	10	5
Complete	Female	USA	North America	English	Yes	Yes	Yes	25-50%	8	3	2
Complete	Female	Switzerland	Europe	Non-English	No	No	No		0		
Complete	Male	Germany	Europe	Non-English	Yes	Yes	Yes	25-50%	20	8	6
Complete	Male	Spain	Europe	Non-English	Yes	No	No	51-75%	11	3	8
Complete	Male	Japan	Other	Non-English	No	No	No	>75%	0		
Complete	Female	Spain	Europe	Non-English	No	No	No		0		
Complete	Male	USA	North America	English	Yes	Yes	Yes	51-75%	21	20	4
Complete	Male	France	Europe	Non-English	No	No	No		0		
Complete	Male	Germany	Europe	Non-English	Yes	No	Yes	25-50%	5	5	6
Complete	Female	Switzerland	Europe	Non-English	Yes	Yes	Yes	25-50%	15	3	6
Complete	Male	USA	North America	English	Yes	No	No	25-50%	12	3	6
Complete	Male	Australia	Other	English	Yes	No	No		5	1	6
Complete	Female	USA	North America	English	Yes	No	No	<25%	4	2	12
Complete	Male	UK	Europe	English	No	No	No		0		
Complete	Male	USA	North America	English	Yes	Yes	Yes	25-50%	4	6	10
Complete	Female	USA	North America	English	Yes	No	No	25-50%	6	3	8
Complete	Male	USA	North America	English	Yes	Yes	Yes	51-75%	25	50	3
Complete	Male	Poland	Europe	Non-English	Yes	Yes	Yes	25-50%	10	10	10
Complete	Male	Finland	Europe	Non-English	Yes	Yes	Yes	51-75%	4	2	8
Incomplete					No	No	No		10		
Complete	Female	USA	North America	English	Yes	No	No	<25%	8	1	6
Complete	Female	UK	Europe	English	Yes	No	No	<25%	1	1	3
Complete	Male	USA	North America	English	Yes	Yes	Yes	<25%	37	4	5
Complete	Male	Germany	Europe	Non-English	Yes	Yes	Yes	51-75%	6	5	5
Complete	Female	Sweden	Europe	Non-English	Yes	Yes	No		6	1	2
Complete	Male	France	Europe	Non-English	Yes	Yes	No	51-75%	18	15	2
Complete	Male	Canada	North America	English	Yes	Yes	Yes	25-50%	3	10	10
Complete	Male	Sweden	Europe	Non-English	No	No	No		3		
Complete	Male	UK	Europe	English	Yes	Yes	Yes	51-75%	10	15	4
Complete	Male	Canada	North America	English	Yes	No	Yes	25-50%	9	5	6
Complete	Male	Mexico	North America	Non-English	Yes	No	Yes	51-75%	18	7	8
Complete	Female	Brazil	Other	Non-English	Yes	No	No	25-50%	19	7	3
Complete	Female	New Zealand	Other	English	Yes	No	No		10	2	5
Complete	Female	Sweden	Europe	Non-English	Yes	Yes	Yes	<25%	7	2	3
Complete	Male	Canada	North America	English	Yes	Yes	No	25-50%	8	5	10
Complete	Male	France	Europe	Non-English	Yes	Yes	Yes	51-75%	17	15	3
Complete	Male	USA	North America	English	Yes	No	No	>75%	4	2	3

Complete	Male	USA	North America	English	Yes	Yes	Yes	25-50%	30	4	6
Complete	Male	Czech Republi	Europe	Non-English	Yes	Yes	No	25-50%	12	5	3
Complete	Male	Poland	Europe	Non-English	Yes	No	No	25-50%	12	3	12
Complete	Male	USA	North America	English	Yes	Yes	Yes	51-75%	12	20	3
Complete	Male	Germany	Europe	Non-English	Yes	Yes	Yes	<25%	8	2	5
Complete	Male	Norway	Europe	Non-English	No	No	No				
Complete	Female	Australia	Other	English	Yes	Yes	No	>75%	3	15	8
Complete	Male	New Zealand	Other	English	Yes	No	Yes	25-50%	29	4	2
Complete	Female	Finland	Europe	Non-English	No	No	No				
Complete	Male	USA	North America	English	Yes	Yes	Yes	51-75%	10	8	10
Complete	Female	USA	North America	English	Yes	Yes	Yes	<25%	7	30	12
Complete	Male	UK	Europe	English	Yes	No	No	<25%	0	10	5
Complete	Male	USA	North America	English	Yes	Yes	No		3	1	4
Complete	Male	Argentina	Other	Non-English	Yes	No	No	25-50%	15	2	48
Complete	Female	Canada	North America	English	Yes	No	No	>75%	3	3	20
Complete	Male	Canada	North America	English	Yes	Yes	Yes	25-50%	13	6	8
Complete	Male	USA	North America	English	Yes	Yes	Yes	51-75%	17	45	1.5
Complete	Male	Australia	Other	English	No	No	No				
Complete	Female	Canada	North America	English	Yes	Yes	Yes	25-50%	37	4	3
Complete	Male	Mexico	North America	Non-English	Yes	No	No	<25%	21	5	6
Complete	Male	Finland	Europe	Non-English	Yes	Yes	Yes	25-50%	4	2	10
Incomplete	Male				Yes	No	No	51-75%	2	2	24
Complete	Female	Canada	North America	English	Yes	Yes	No	51-75%	9	10	3
Complete	Male	Canada	North America	English	Yes	Yes	Yes	51-75%	13	10	8
Complete	Male	UK	Europe	English	Yes	No	No	25-50%	6	3	4
Complete	Male	Switzerland	Europe	Non-English	Yes	No	No	25-50%	5	5	6
Complete	Female	USA	North America	English	Yes	Yes	Yes	51-75%	4	3	12
Complete	Male	Germany	Europe	Non-English	Yes	No	No	51-75%	7	5	12
Complete	Female	Israel	Other	Non-English	Yes	Yes	No	25-50%	7	6	15
Complete	Female	USA	North America	English	No	No	No				
Complete	Male	France	Europe	Non-English	Yes	Yes	Yes	25-50%	21	8	1
Complete	Male	Sweden	Europe	Non-English	Yes	Yes	Yes	25-50%	19	50	1.5
Complete	Male	Spain	Europe	Non-English	Yes	No	Yes	25-50%	17	10	8
Complete	Male	USA	North America	English	Yes	Yes	Yes	>75%	13	10	3
Complete	Male	Germany	Europe	Non-English	Yes	Yes	No	25-50%	10	1	8
Complete	Female	Canada	North America	English	No	No	No				
Complete	Male	France	Europe	Non-English	Yes	Yes	Yes	25-50%	8	15	8
Complete	Male	Germany	Europe	Non-English	Yes	No	No	25-50%	19	8	2
Complete	Male	USA	North America	English	Yes	No	No	<25%	0	1	3.5
Complete	Male	France	Europe	Non-English	Yes	No	Yes	51-75%	8	12	8
Complete	Male	UK	Europe	English	No	No	No		0		
Complete	Male	USA	North America	English	Yes	Yes	Yes	51-75%	9	20	2.5
Complete	Male	Czech Republi	Europe	Non-English	Yes	No	No	25-50%	9	6	8
Complete	Male	Canada	North America	English	Yes	Yes	Yes	51-75%	9	35	3
Complete	Female	UK	Europe	English	No	No	No		6		

Complete	Male	USA	North America	English	No	No	No		4		
Complete	Female	USA	North America	English	Yes	No	No	51-75%	5	4	8
Complete	Female	USA	North America	English	Yes	No	Yes	25-50%	12	6	8
Complete	Male	USA	North America	English	Yes	Yes	Yes	25-50%	30	15	5
Complete	Male	USA	North America	English	Yes	No	No	<25%	11	5	5
Complete	Male	Netherlands	Europe	Non-English	No	No	No		2		
Complete	Male	Norway	Europe	Non-English	Yes	Yes	No	25-50%	9	4	4
Complete	Male	Israel	Other	Non-English	Yes	Yes	Yes	25-50%	14	2	15
Complete	Male	USA	North America	English	Yes	No	No	>75%	6	2	72
Complete	Female	UK	Europe	English	Yes	No	No	<25%	18	7	6
Complete	Male	USA	North America	English	Yes	Yes	Yes	51-75%	17	8	3
Complete	Male	Australia	Other	English	Yes	Yes	Yes	51-75%	22	3	2
Complete	Male	USA	North America	English	Yes	No	Yes	25-50%	16	5	16
Complete	Male	Canada	North America	English	Yes	Yes	No	25-50%	4	15	2
Complete	Female	USA	North America	English	Yes	No	No	<25%	6	18	2
Complete	Male	USA	North America	English	Yes	Yes	Yes	51-75%	19	25	6
Complete	Male	USA	North America	English	No	No	No		0		
Complete	Female	Finland	Europe	Non-English	Yes	Yes	Yes	25-50%	9	5	12.5
Complete	Female	Switzerland	Europe	Non-English	Yes	Yes	Yes	25-50%	7	5	6
Complete	Male	Norway	Europe	Non-English	Yes	Yes	Yes	<25%	17	10	8
Incomplete	Male				Yes	No	Yes	25-50%	11	15	6
Complete	Female	USA	North America	English	No	No	No		0		
Complete	Male	USA	North America	English	No	Yes	No		3		
Complete	Female	USA	North America	English	Yes	Yes	Yes	<25%	6	6	6
Complete	Male	USA	North America	English	Yes	Yes	No	<25%	6	3	2.5
Complete	Female	USA	North America	English	Yes	No	No	25-50%	3	4	3
Complete	Male	USA	North America	English	Yes	Yes	No		1		
Complete	Male	USA	North America	English	Yes	Yes	Yes	51-75%	20	10	6
Complete	Male	USA	North America	English	Yes	No	No	25-50%	24	20	3
Complete	Male	USA	North America	English	Yes	No	No	<25%	4	5	15
Complete	Female	New Zealand	Other	English	Yes	Yes	Yes	<25%	4	2	10
Complete	Male	USA	North America	English	No	No	No				
Complete	Male	USA	North America	English	Yes	Yes	No	25-50%	10	1	8
Complete	Male	USA	North America	English	Yes	Yes	Yes	25-50%	19	5	2
Complete	Male	USA	North America	English	Yes	No	Yes	51-75%	9	12	8
Complete	Male	USA	North America	English	Yes	Yes	Yes	51-75%	16	40	6
Complete	Male	USA	North America	English	Yes	Yes	No	51-75%	6	3	3
Complete	Female	USA	North America	English	Yes	Yes	Yes	<25%	2	14	5
Complete	Female	USA	North America	English	Yes	No	No	51-75%	6	4	8
Complete	Male	USA	North America	English	Yes	No	No	25-50%	6	10	20
Complete	Male	USA	North America	English	Yes	No	Yes	25-50%	21	6	8
Complete	Female	USA	North America	English	Yes	No	No	25-50%	23	4	6
Complete	Male	USA	North America	English	Yes	No	No	25-50%	39	25	2
Complete	Male	USA	North America	English	Yes	No	Yes	25-50%	26	4	2
Complete	Female	USA	North America	English	Yes	Yes	Yes	51-75%	25	15	4

Complete	Male	USA	North America	English	Yes	Yes	Yes	25-50%	16	20	3
Complete	Male	USA	North America	English	Yes	No	No	<25%	4	3	10
Complete	Male	Kenya	Other	English	Yes	Yes	No		5	2.5	2
Complete	Female	USA	North America	English	Yes	Yes	Yes	51-75%	8	8	5
Complete	Male	USA	North America	English	Yes	Yes	Yes	>75%	15	8	4
Complete	Male	USA	North America	English	Yes	Yes	No	<25%	26	2	3
Complete	Male	USA	North America	English	Yes	Yes	Yes	>75%	41	5	2
Complete	Female	USA	North America	English	Yes	No	Yes	51-75%	22	25	12
Complete	Female	USA	North America	English	No	No	No				
Complete	Male	USA	North America	English	Yes	No	No	51-75%	0	5	4
Complete	Male	USA	North America	English	Yes	Yes	Yes	51-75%	24	5	10
Complete	Male	USA	North America	English	Yes	Yes	Yes	25-50%	33	2	5
Complete	Male	Israel	Other	Non-English	Yes	Yes	No	25-50%	3		10
Complete	Female	USA	North America	English	Yes	No	Yes	25-50%	1	5	24
Complete	Female	USA	North America	English	No	No	No		2		
Complete	Male	USA	North America	English	Yes	Yes	Yes	<25%	15	6	5
Complete	Male	USA	North America	English	Yes	Yes	Yes	51-75%	5	2	16
Complete	Female	USA	North America	English	Yes	Yes	No	51-75%	8	4	3
Complete	Male	USA	North America	English	Yes	Yes	Yes	51-75%	25	10	5
Complete	Male	USA	North America	English	Yes	Yes	Yes	25-50%	10	6	6
Complete	Male	Canada	North America	English	Yes	Yes	Yes	25-50%	42	1	2
Complete	Female	USA	North America	English	No	No	No				
Complete	Male	USA	North America	English	Yes	No	Yes	25-50%	3	4	6
Complete	Female	USA	North America	English	Yes	No	Yes	<25%	7	2	20
Complete	Male	USA	North America	English	Yes	Yes	Yes	51-75%	10	11	6
Complete	Female	Australia	Other	English	Yes	No	Yes	>75%	14	3	3
Complete	Female	USA	North America	English	Yes	Yes	Yes	25-50%	1	4	3
Complete	Male	USA	North America	English	Yes	No	No		6	4	8
Complete	Female	USA	North America	English	No	No	No		1		
Complete	Male	USA	North America	English	Yes	No	No		0	1	3
Complete	Female	USA	North America	English	Yes	Yes	No	51-75%	0	4	10
Complete	Male	USA	North America	English	Yes	Yes	No	25-50%	8	1	4
Complete	Male	USA	North America	English	Yes	No	No	25-50%	4	12	3
Complete	Female	USA	North America	English	Yes	Yes	Yes	25-50%	22	15	8
Complete	Female	USA	North America	English	Yes	Yes	Yes	>75%	8	10	8
Complete	Male	Canada	North America	English	Yes	Yes	Yes	25-50%	2	12	8
Complete	Female	USA	North America	English	Yes	No	No	25-50%	2	1	5
Complete	Female	USA	North America	English	Yes	Yes	No		0	1	10
Complete	Male	USA	North America	English	No	No	No		3		
Complete	Male	USA	North America	English	Yes	No	No	25-50%	0	1	4
Complete	Male	USA	North America	English	Yes	Yes	Yes	51-75%	13	10	4
Complete	Female	USA	North America	English	Yes	Yes	Yes	51-75%	13	6	4
Complete	Male	USA	North America	English	No	No	No		0		
Complete	Female	USA	North America	English	Yes	No	No	25-50%	17	2	6
Complete	Male	USA	North America	English	Yes	Yes	Yes	<25%	26	10	3

Complete	Male	USA	North America	English	Yes	No	Yes	>75%	9	5	3
Complete	Female	Finland	Europe	Non-English	Yes	No	No	<25%	7	2	30
Complete	Male	USA	North America	English	Yes	Yes	Yes	<25%	9	2	10
Complete	Male	Switzerland	Europe	Non-English	Yes	Yes	No	25-50%	3	7	10
Complete	Female	Spain	Europe	Non-English	Yes	No	No	25-50%	5	3	10
Complete	Male	France	Europe	Non-English	Yes	No	No	<25%	8	5	3
Complete	Female	Taiwan	Other	Non-English	No	Yes	No		17		
Complete	Male	USA	North America	English	Yes	Yes	Yes	25-50%	21	5	3
Complete	Male	USA	North America	English	Yes	Yes	Yes	51-75%	5	10	6
Complete	Female	USA	North America	English	Yes	Yes	Yes	<25%	7	2	15
Complete	Female	Sweden	Europe	Non-English	Yes	Yes	Yes	25-50%	7	4	10
Complete	Male	France	Europe	Non-English	Yes	No	No	>75%	2	5	5
Incomplete	Male				Yes	No	No	25-50%	40	6	4
Complete	Female	USA	North America	English	No	Yes	No		0		
Complete	Male	USA	North America	English	Yes	No	No	51-75%	10	2	2
Complete	Female	Norway	Europe	Non-English	Yes	No	No	51-75%	19	2	35
Complete	Male	Germany	Europe	Non-English	Yes	No	No		2		10
Complete	Male	Germany	Europe	Non-English	Yes	No	No	51-75%	6	4	8
Complete	Male	USA	North America	English	Yes	No	No	25-50%	5	5	10
Complete	Male	Italy	Europe	Non-English	Yes	No	No	51-75%	25	24	0.75
Complete	Male	USA	North America	English	Yes	Yes	Yes	25-50%	19	5	7
Complete	Male	Germany	Europe	Non-English	Yes	No	No	51-75%	15	10	12
Complete	Female	USA	North America	English	Yes	Yes	Yes	25-50%	8	6	5
Complete	Male	USA	North America	English	Yes	No	No	51-75%	5	1	3
Complete	Male	USA	North America	English	No	No	No				
Complete	Male	Argentina	Other	Non-English	Yes	No	No	<25%	12	4	4
Complete	Male	USA	North America	English	Yes	Yes	Yes	25-50%	18	5	6
Complete	Male	USA	North America	English	Yes	No	No		0		
Complete	Male	USA	North America	English	Yes	No	Yes	25-50%	17	10	2
Complete	Male	USA	North America	English	Yes	No	Yes	25-50%	27	5	4
Complete	Female	USA	North America	English	Yes	No	No	51-75%	16	5	6
Complete	Male	Denmark	Europe	Non-English	Yes	No	No	51-75%	19	5	30
Complete	Male	USA	North America	English	Yes	No	No	25-50%	4	2	8
Complete	Male	USA	North America	English	Yes	No	No			2	4
Complete	Female	USA	North America	English	Yes	Yes	Yes	<25%	14	4	10
Complete	Male	USA	North America	English	Yes	No	No	25-50%	12	3	16
Complete	Male	USA	North America	English	Yes	No	No	<25%	3	12	12
Complete	Female	USA	North America	English	Yes	Yes	No		9	2	6
Complete	Male	USA	North America	English	Yes	No	Yes	25-50%	11	10	20
Complete	Male	USA	North America	English	Yes	No	Yes	<25%	18	5	3
Complete	Male	Canada	North America	English	Yes	Yes	Yes	51-75%	5	3	15
Complete	Male	USA	North America	English	Yes	Yes	No	>75%	9	15	3
Complete	Male	USA	North America	English	Yes	No	No	25-50%	0	4	10
Complete	Male	USA	North America	English	Yes	No	Yes	<25%	29	4	4
Complete	Female	USA	North America	English	Yes	Yes	Yes	<25%	11	10	3

Complete	Male	USA	North America	English	No	No	No		2		
Complete	Female	USA	North America	English	Yes	No	No	<25%	5	3	5
Complete	Female	USA	North America	English	No	No	No		0		
Complete	Female	Canada	North America	English	Yes	No	No	51-75%	3	1.5	6.5
Complete	Male	USA	North America	English	Yes	No	No	<25%	12	5	5
Complete	Female	USA	North America	English	Yes	No	No	<25%	3	2.5	10
Complete	Female	USA	North America	English	Yes	Yes	No	<25%	5	2	4
Complete	Male	USA	North America	English	No	No	No		0		
Complete	Male	USA	North America	English	Yes	Yes	Yes	>75%	0	2	4
Complete	Male	USA	North America	English	Yes	Yes	Yes	25-50%	2	5	1.5
Complete	Female	USA	North America	English	Yes	No	No	>75%	6	1	10
Complete	Male	USA	North America	English	Yes	No	Yes	<25%		2	4
Complete	Female	USA	North America	English	Yes	No	No	25-50%	24	8	10
Complete	Male	USA	North America	English	Yes	No	No	<25%	2	2	4
Complete	Female	USA	North America	English	Yes	Yes	No		2	1	4
Complete	Male	USA	North America	English	Yes	No	Yes	<25%	6	1	6
Complete	Male	USA	North America	English	Yes	No	No	25-50%	4	1	10
Complete	Female	USA	North America	English	Yes	Yes	No	51-75%	6	6	3
Complete	Male	USA	North America	English	Yes	No	Yes	51-75%	10	4	10
Complete	Male	USA	North America	English	Yes	Yes	Yes	25-50%	28	2	4
Complete	Female	USA	North America	English	Yes	No	No	25-50%	2	3	3
Complete	Male	USA	North America	English	Yes	Yes	Yes	<25%	4	3	6
Complete	Female	USA	North America	English	Yes	Yes	Yes	51-75%	11	4	3
Complete	Male	USA	North America	English	Yes	Yes	Yes	51-75%	5	10	6
Complete	Female	USA	North America	English	Yes	No	No	25-50%	11	5	10
Complete	Male	USA	North America	English	Yes	Yes	Yes	25-50%	24	15	3
Complete	Male	USA	North America	English	No	No	No		2		
Complete	Male	USA	North America	English	Yes	Yes	No	51-75%	0	2	4
Complete	Male	USA	North America	English	Yes	No	No	51-75%	3	4	8
Complete	Male	USA	North America	English	Yes	No	No	25-50%	9	4	5
Complete	Male	USA	North America	English	Yes	No	No	<25%	11	2	16
Complete	Female	USA	North America	English	Yes	Yes	Yes	<25%	2	2	4
Complete	Male	USA	North America	English	No	No	No		1		
Complete	Female	USA	North America	English	Yes	No	No			2	6
Complete	Male	USA	North America	English	No	Yes	No				
Complete	Male	USA	North America	English	Yes	Yes	Yes	25-50%	5	5	5
Complete	Female	USA	North America	English	Yes	No	Yes	<25%	9	8	40
Complete	Female	USA	North America	English	Yes	No	Yes	25-50%	19	5	6
Complete	Female	USA	North America	English	No	No	No		2		
Complete	Male	Canada	North America	English	Yes	Yes	Yes	>75%	6	5	16
Complete	Female	USA	North America	English	Yes	Yes	Yes	51-75%	11	10	3
Complete	Female	USA	North America	English	Yes	No	No	25-50%	1	3	6
Complete	Female	USA	North America	English	Yes	No	Yes	25-50%	18	5	6
Complete	Female	USA	North America	English	Yes	No	No	25-50%	26	2	6
Complete	Male	USA	North America	English	Yes	No	No	<25%	17	2	10

Complete	Male	USA	North America	English	Yes	No	No	51-75%	10	1	4
Complete	Female	USA	North America	English	Yes	Yes	No	<25%	10	3.5	4
Complete	Female	Australia	Other	English	Yes	No	No	<25%	4	2	3
Complete	Female	Canada	North America	English	Yes	No	No	51-75%	17	7	3
Complete	Male	Australia	Other	English	Yes	Yes	No		14	1	8
Complete	Male	Australia	Other	English	Yes	Yes	Yes	51-75%	34	100	1
Complete	Female	USA	North America	English	Yes	No	No	<25%	8	2	6
Complete	Male	USA	North America	English	Yes	Yes	No	>75%	7	6	10
Complete	Male	Norway	Europe	Non-English	Yes	Yes	Yes	25-50%	12	12	6
Complete	Male	Germany	Europe	Non-English	Yes	No	No		6	1	3
Complete	Male	USA	North America	English	Yes	No	Yes	25-50%	3	6	16
Complete	Male	USA	North America	English	Yes	No	Yes		28	4	4
Complete	Female	USA	North America	English	No	No	No		5		
Complete	Female	USA	North America	English	Yes	No	No		11	1	10
Complete	Male	USA	North America	English	No	Yes	No	<25%	2		
Complete	Female	Germany	Europe	Non-English	Yes	Yes	No	25-50%	3	2	6.5
Complete	Male	Brazil	Other	Non-English	Yes	No	Yes	51-75%	18	6	2
Complete	Female	USA	North America	English	Yes	Yes	Yes	51-75%	10	5	10
Complete	Male	Canada	North America	English	Yes	No	Yes	25-50%	14	12	5
Complete	Male	Italy	Europe	Non-English	Yes	No	No	25-50%	3	6	6
Complete	Female	UK	Europe	English	Yes	No	No	<25%	2	2.5	5
Complete	Male	Netherlands	Europe	Non-English	Yes	Yes	Yes	<25%	17	12	5
Complete	Female	South Africa	Other	English	Yes	Yes	Yes	>75%	8	10	10
Complete	Male	UK	Europe	English	No	No	No				
Incomplete	Female	USA	North America	English	Yes	No	No	51-75%	4	3	6
Complete	Male	UK	Europe	English	Yes	Yes	Yes	25-50%	12	3	10
Complete	Male	Mexico	North America	Non-English	Yes	No	No	25-50%	16	8	12
Complete	Female	Israel	Other	Non-English	Yes	Yes	Yes	25-50%	12	6	3
Complete	Male	USA	North America	English	Yes	No	No	<25%	19	2	4
Complete	Female	Portugal	Europe	Non-English	No	No	No		1		
Complete	Male	UK	Europe	English	Yes	Yes	Yes	51-75%	9	20	5
Complete	Female	USA	North America	English	Yes	No	Yes		0	4	4
Incomplete	Male				No	No	Yes				
Complete	Male	UK	Europe	English	Yes	No	No	<25%	10	12	2
